# Identification of candidate genes involved in salt stress response at germination and seedling stages by QTL mapping in upland cotton

**DOI:** 10.1093/g3journal/jkac099

**Published:** 2022-04-26

**Authors:** Anhui Guo, Ying Su, Hushuai Nie, Bin Li, Xingkun Ma, Jinping Hua

**Affiliations:** Laboratory of Cotton Genetics, Genomics and Breeding/Key Laboratory of Crop Heterosis and Utilization of Ministry of Education, Ministry of Education/Beijing Key Laboratory of Crop Genetic Improvement, College of Agronomy and Biotechnology, China Agricultural University, Beijing 100193, China

**Keywords:** salt stress, seed germination, seedling emergence, quantitative trait loci, upland cotton

## Abstract

Salinity is a major abiotic stress at critical stages of seed germination and seedling establishment. Germination rate (GR) and field emergence rate (FER) are the key traits that determine the basic number of plants stand under field conditions. To explore molecular mechanisms in upland cotton under salt stress, a population of 177 recombinant inbred lines, and their parents were evaluated for seed germination traits (GP, germination potential; GR; FW, fresh weight; DW, dry weight; GL, germinal length) and seedling traits (FER; SH, seedling height; NL, number of main stem leaves) in 2016–2018. Based on the linkage map contained 2,859 single nucleotide polymorphism and simple sequence repeat markers, traits under salt stress (E1) and normal conditions (E2), and in the converted relative index (R-value) dataset of 3 years’ trials were used to map quantitative trait loci (QTL). A total of 3 QTL and 2 clusters were detected as salt-tolerant QTL. Three QTL (*qGR-Chr4-3*, *qFER-Chr12-3*, and *qFER-Chr15-1*) were detected under salt stress conditions and *R*-value dataset, which explained variance of phenotype 9.62–13.67%, and 4.2–4.72%, 4.75–8.96%, respectively. Two clusters (Loci-Chr4-2 and Loci-Chr5-4) harboring the QTL for 4 germination traits (GR, FER, GL, and NL) and 6 seedling traits (GR, FER, DW, FW, SH, and NL) were detected related under salt stress. A total of 691 genes were found in the candidate QTL or clusters. Among them, 4 genes (*Gh_A04G1106, Gh_A05G3246, Gh_A05G3177*, and *Gh_A05G3266*) showed expression differences between salt-sensitive and -tolerant lines under salt stress conditions, and were assigned as candidate genes in response to salt stress. The consistent salt-tolerance QTL identified in both germination and seedling stages will facilitate novel insights into effective utilization of cotton genetic resources.

## Introduction

High salinity is one of the major abiotic stresses limiting plants growth and decreasing the yield (Parida and Das 2005; Ismail and Horie 2017). Salinity affects 1 billion hectares of arid and semiarid areas globally, and soil salinity will become progressively more severe due to climatic changes, excessive irrigation, and excessive fertilization (Han *et al.* 2015).

Salt stress inhibits osmotic and ionic homeostasis at a cellular level, further inhibits photosynthesis and reduces cellular energy, and eventually results in redox imbalance during seed imbibition, germination, and root elongation (Liang *et al.* 2018). Many factors can affect the salt tolerance evaluation of plants, including the phase of plant growth, the duration of exposure to salinity, the salt concentration, the plant species, and the method of evaluation (Higbie *et al.* 2010). Plants at germination, emergence, and seedling stages are more susceptible to salt stress than other stages (He *et al.* 2019). The reduced germination potential (GP), fresh mass, and vigor index lead to delayed germination and fewer germinated seeds (Ahmad *et al.* 2002). At seedling stage, root is the first organ exposed to salt stress (Galvan-Ampudia *et al.* 2013; Robbins *et al.* 2014). Salinity generally disrupts the cell cycle activity at the root meristems, resulting in reduced root development and afterwards plant growth in cotton (West *et al.* 2004). To evaluate the performance and tolerance in upland cotton plants under salt stress, germination, and seedling characteristics were the most viable criteria (Ahmad *et al.* 2002). Most common salt tolerance traits used in the quantitative trait loci (QTL) mapping under salt stress include GP, germination rate (GR), and root growth-related traits (shoot length, root length, root fresh weight, and root dry weight) (Du *et al.* 2016; Diouf *et al.* 2017; Yuan *et al.* 2019; Sikder *et al.* 2020). Traits such as plant height, shoot dry weight, and root dry weight are sensitive to a salinity of 150 mM NaCl at the seedling stage of 5–6 leaves in cotton (Du *et al.* 2016).

Upland cotton (*Gossypium hirsutum* L., AADD, 2*n* = 52), an important cash crop, is considered as a pioneer crop in saline-alkali land. The release of genome data of upland cotton (Li *et al.* 2015; Zhang *et al.* 2015; Hu *et al.* 2019; Wang *et al.* 2019; Yang *et al.* 2019; Chen *et al.* 2020; Huang *et al.* 2020) has greatly facilitated researches to develop high-density linkage map construction. Different strategies were used to construct genetic maps (Jamshed *et al.* 2016; Zhang *et al.* 2016). Currently, array-based single nucleotide polymorphism (SNP) markers are the effective genotyping method in cotton due to their low cost and limited reliance on computational resources (Jia *et al.* 2016). A newly released genetic map harbored 3,009 polymorphic SNP markers and covered a total genetic distance of 4,983.73 cM with an average marker interval of 1.66 cM (Zhu *et al.* 2021). Futhermore, a high-density linkage map containing 6,187 bin markers spanning 4,478.98 cM with an average marker interval of 0.72 cM has been constructed (Gu *et al.* 2020).

QTL mapping is an effective strategy for quantitative trait research, and has been widely used in agriculture to map a set of traits, such as salt tolerance (Ashraf and Foolad 2013). To date, numerous QTL for seed germination and seedling growth under salt stress conditions have been identified. A total of 8 clusters controlled at least 2 salt-tolerance traits located on chromosomes (Chr) A02, A06, A12, D03, D06, D08, and D13 were identified using a F_2:3_ population of 277 lines derived from an intraspecific cross between 2 upland cotton accessions CCRI35 and NH. In these clusters, 12 genes were highly expressed in response to salt treatment (Diouf *et al.* 2017). Moreover, 11 consistent QTL have been identified in 8 chromosomes with *qRL-Chr16-1* of root length, explaining the variance of phenotype ranged from 11.97% to 18.44% (Oluoch *et al.* 2016). Furthermore, 13 QTL controlled dry root weight on 9 chromosomes have been detected under salt stress treatment in an introgressed recombinant inbred line (RIL) population of upland cotton (Abdelraheem *et al.* 2018). The QTL *qSalt-A04-1* associated with salt tolerance in the seed germination stage has been characterized via using an RIL upland cotton population of 577 lines (Gu *et al.* 2021). Several important SNPs responding to salt stress at seedling stage have also been identified in upland cotton, for instance, 23 SNPs, which represented 7 genomic regions in Chr A01, A10, D02, D08, D09, D10, and D11, have been found to be associated with the relative survival rate (RSR) and salt tolerance level (STL) by using a natural population comprising 713 upland cotton accessions. Of which, 6 genes (*Gh_D10G1888*, *Gh_D02G0060*, *Gh_D09G0943*, *Gh_D10G1821*, *Gh_D09G0958*, and *Gh_A10G1756*) related to salt tolerance were validated (Gu *et al.* 2021). In addition, a total of 4 genes (*GhPIP3A, GhSAG29, GhTZF4*, and *GhTZF4a*) located on chromosome D01 were assigned as candidate genes associated with relative GR under salt stress conditions (Sun *et al.* 2019).

Seedling number is critical for population construction in commercial production all over the world. However, few studies have been reported on the effects of salt stress at the seedling stage in upland cotton under natural saline conditions. In the present study, 8 phenotypic traits related to salt tolerance were evaluated and data were collected from 177 RILs derived from a cotton hybrid “Xinza 1” (Shang *et al.* 2015, 2016). The genetic linkage map consisting of 2,859 bins was used to analyze salt tolerance related QTL (Guo *et al.* 2021). The objective of this research was to identify candidate genes in response to salt stress from the QTL and provide valuable insights into the mechanisms underlying salt stress. The findings of this study provide valuable insights in developing salt-tolerant cultivars and screening salt stress-responsive genes.

## Materials and methods

### Plant materials

The RIL population derived from a hybrid “Xinza 1” (GX1135 × GX100-2) was employed by single seed descent method in upland cotton (Shang *et al.* 2016), including 177 lines. The control set included GX1135, “Xinza 1” F_1_, GX100-2, and a commercial hybrid “Ruiza 816” used as competition control (Ma *et al.* 2020; Guo *et al.* 2022). A total of 177 RILs of F_16_–F_18_ generations were assigned in the field salt stress experiment. One hundred forty-four RILs of F_16_ generations were used in the indoor germination experiment (Supplementary Table 1).

### Indoor seed germination test and phenotypic evaluation

The salt tolerance experiment was carried out under 150 mM NaCl and control conditions, respectively, and both with a 12–12 h day/night photoperiod, corresponding to a temperature of 28°C ± 1°C. A total of 300 full and robust tufted seeds were selected for the salt tolerance experiment, of which 50 seeds for each repeat were soaked for 12 h. For salt stress treatment, the seeds were soaked in 150 mM NaCl solution for 12 h, then wrapped in the filter paper and irrigated with 150 mM NaCl solution to maintain water levels (2 L) at germination stage. Under normal conditions, deionized water was used during the test period. The germination test was carried out by using the method of filter paper roll germination. A randomized complete design with 3 biological replicates was adopted in salt stress or normal conditions.

To analyze the traits, we performed indoor germination experiments in which cotton seeds were sown enrolling within the filter papers. On the third day after sowing, we investigated the trait GP (%). On day 7 after sowing, we investigated the traits GR (%), germinal length (GL, cm), fresh weight (FW, g), and dry weight (DW, g).

### Field arrangement and traits evaluation

Two field trials under salt and control conditions were conducted in 2017, 2018, and 2019 in Quzhou Experimental Station of China Agricultural University, Handan City, Hebei Province (36°78′N, 114°92′E).

Two independent field trials were arranged in neighboring fields following a randomized complete block design with 2 replications each trial on 2017 May 2 (spring of 2017, 2017t1), 2017 August 22 (summer of 2017, 2017t2), 2018 April 29 (spring of 2018, 2018), 2019 May 6 (spring of 2019, 2019t1), and 2019 July 6 (summer of 2019, 2019t2). For the field salt tolerance experiments, field emergence rate (FER), seedling height (SH), and number of main stem leaves (NL) were determined at the seedling stage. The FER was investigated on 2017 May 16, 2017 August 31, 2018 May 17, 2019 May 28, 2019 July 30, respectively. The SH and NL traits were investigated on 2019 June 17 and 2019 August 16.

A total of 362 plots with 2 rows (22 individuals each row) were conducted, respectively. Two repeats of 177 RILs (F_16_–F_18_) were planted together with 2 control sets (GX1135, GX100-2, “Xinza 1,” and “Ruiza 816”). Every 2-row plot was 80 and 60 cm row spacing with plot length of 2.4 m, following 0.7 m pavement apart for field experiments.

For salt stress treatment, shallow saline groundwater with the concentration of 5 g/L (85 mM) saline was used to irrigate the field twice before sowing in 2017 and 2018 (Guo *et al.* 2021), and shallow saline groundwater with the concentration of 7 g/L (120 mM) saline was used to irrigate the field in 2019. For the control treatment, regular irrigation was performed when needed. Field management followed the local standard field practices. The normal irrigation treatment included regular irrigation consistent with the standard agronomic practices for the conditions in that year.

### Soil samples collected and soil property measurement

Soil samples were collected from the depth of 0 to 20 and 20 to 40 cm after sowing. To cover the experiment area, sampling points were chosen every 15 m from north to south in the experiment field. Three soil samples collected for each sample site were mixed into 1 sample for soil quality determination (Guo *et al.* 2021). Soil saturated paste extracts (1:2 by weight) were prepared to measure the electrical conductivity (EC) and total content of water-soluble salt (ρ) of the samples (Rhoades 1996; Shang *et al.* 2016). Three independent repeats were detected for each sample.

### Genetic linkage map construction

Linkage map analysis was conducted using Join Map 4.0. The adjacent markers from the same parent were recorded as 1 bin (Xie *et al.* 2010). A total of 2,859 bins which including 330 simple sequence repeat (SSR) markers and 2,529 SNPs were distributed on the new linkage map (Supplementary Fig. 2). The genetic map covered 2,133.53 cM of cotton genome with an average interval of 0.785 cM (Guo *et al.* 2021). Among the 26 linkage groups, the average bin spacing was 5.979 cM, and the range of interval on 26 chromosomes was 3.647–10.361 cM, and only 1 gap larger than 10 cM existed on Chr D05.

### Data analysis and QTL mapping

Three datasets of (1) salt stress conditions (E1); (2) normal conditions (E2); and (3) the converted relative index (*R*-value) were used in the present study. The original data of 8 salt-tolerant-related traits were obtained from the trials under E1 and E2, respectively. The R-value dataset of 8 salt-related traits were calculated using the following formula: (phenotypic effects value under E1/phenotypic effects value under E2) × 100%. To estimate phenotypic traits across multiple environments, the phenotypic values of the accessions from multiple environments were estimated via best linear unbiased prediction based on a linear model fitted with the lme4 R package (Technow *et al.* 2012). Analysis of variance (ANOVA) was conducted for all traits separately for estimating variance components for evaluation of the significance of genotypes and environmental effects and their interactions in the RIL population. SD, coefficients of variation, ANOVA, and correlation analyses were estimated using the software SPSS (Version 21.0, Chicago). The broad-sense heritability percentage, *h*^2^_B_, was calculated for each trait using the formula (Singh *et al.* 2017):

$$h_{B}^{2}=\frac{\sigma_{G}^{2}}{\sigma_{G}^{2}+\frac{\sigma_{G}^{2}\times E}{n_{e}}+(\frac{\sigma_{E}^{2}}{n_{e}n_{r}})}\times 100\%.$$

With σ^2^_G_ is the genotypic variance; σ^2^_G×E_ is genotype × environment interaction; σ^2^_E_ is phenotypic variance (PV); *n*_e_ is the number of environments, and *n*_r_ is the number of replications for each environment.

QTL mapping and the genetic effect values at single-locus level were conducted by QTL Cartographer software (Version 2.5) using composite interval mapping (CIM) method (Wang *et al.* 2007). We set parameter in the confidence interval of 95% with the CIM method for QTL mapping. The threshold of LOD values was estimated after 1,000 permutations tests to declare a significant QTL with a significance level of *P < *0.05, whereas QTL in another trial with LOD of at least 2.0 was considered as common QTL (Liang *et al.* 2013; Shang *et al.* 2016).

### Gene function annotation and enrichment analysis

Genes located in the confidence intervals of the candidate QTL were fetched from the CottonGen (https://www.cottongen.org) by using their positions of flanking markers in *G. hirsutum* TM-1 genome (Zhang *et al.* 2015) and considered as the candidate genes. Gene ontology (GO) enrichment and KEGG pathway analysis were carried out for all candidate genes. The GO enrichment was performed on BMK Cloud platform (www.biocloud.net).

### RNA sequencing and analysis

The parents of XZ1 (female GX1135 and male GX100-2) were used for RNA-seq analysis. Seeds were germinated in the sand and then the identical seedlings were transferred into modified 1/2 Hoagland solution. The seedlings were planted in the 1/2 Hoagland solution containing 150 mM NaCl at the 3-leaf stage (Guo *et al.* 2015). Plants were grown at 28°C/20°C, and a photoperiod of 14 h light/10 h dark. Roots of these seedlings were sampled at 1, 3, 12, and 48 h after salt stress treatment, and frozen with liquid nitrogen and stored at −80°C for use.

Total RNA was extracted from the roots of GX1135 and GX100-2 seedlings using a modified cetyltrimethyl ammonium bromide method (Zhao *et al.* 2012), which were treated with 150 mM NaCl solution for 0, 1, 3, 12, and 48 h, respectively, and with 3 biological replicates. RNA-seq libraries were sequenced on Illumina Hiseq 2500 platform (Biomarker Technology Corporation, Beijing, China).

The clean reads of each library were aligned to the reference genome using TopHat software (Version 2.0.1). Gene expression levels were calculated by Cufflinks (Version 2.2.1) of fragments per kilobase of exon per million mapped reads (FPKM) value (Trapnell *et al.* 2012). Differentially expressed genes (DEGs) were detected using DEGseq (Wang *et al.* 2010) with *P*-value <0.01 and fold change (salt treated cv 0 h) >2, which was performed on the BMK Cloud platform (www.biocloud.net).

### RNA extraction and gene expression validation

Total RNA was isolated from the leaf of salt-tolerant lines (T), and salt-sensitive lines (S) under salt stress conditions and normal conditions (Zhao *et al.* 2012), respectively. To validate the potential function in salt stress response, the expression patterns of candidate genes were verified with qRT-PCR using RNA of leaf in salt-tolerant lines and salt-sensitive lines. Gene relative expression level was calculated with 2^−^^ΔΔCt^ method (Livak and Schmittgen 2001). Primers for the qRT-PCR analysis were listed in Supplementary Table 2. Three independent replicates were performed for each sample. *GhUBQ7* gene was used as a reference gene.

## Results

### Phenotypic performance under salt stress condition and normal condition

To ensure the effectiveness of salt stress in the field trial, the EC and ρ of soil samples in the experimental area were measured (Supplementary Table 3). The average ρ of soil samples in the depth of 0–20 cm collected during the sowing period in 2018 were identified as mild-soil salinization (1.67 g/kg) in normal condition and moderate soil salinization (2.08 g/kg) in saline soil, respectively. The average ρ of soil in 20–40 cm depth in 2018 was slightly salinized (0.98 g/kg) under E2 and slightly salinized (1.66 g/kg) under E1. According to the grading standard of saline soil based on EC, the soil samples collected in the depth of 0–20 cm (0.88–0.95 g/kg) was nonsaline soil, while the highly saline soil (4.37 g/kg, 2019t2) or moderately salinized soil (3.97 μs/cm, 2019t1) was collected in the depth of 20–40 cm. The results indicate that there was a difference in salt concentration between the 2 experiments after saline irrigation.

Salt stress significantly inhibited the germination and seedling formation of cotton in both fields and laboratory experiments (Supplementary Figs. 3–6). In most cases, the difference between the 2 parents was significant under E2, but not significant under E1 (Supplementary Fig. 1 and Supplementary Table 4). The phenotype of the male parent GX100-2 was greater than that in the female parent GX1135 for the 8 salt-tolerant related traits under E1 or E2, while there were exceptions such as FER under E1 in 2017t2, NL under E1 and E2 in 2019t2, SH under E1 in 2019t2 and under E2 in 2019t1, GL under E2. In conclusion, the salt tolerance of male parent GX100-2 is better than that of female parent GX1135.

As for RIL population, all traits varied widely among the population (CV = 28.58–112.78% under E1, 6.23–50.78% under E2, and 9.07–192.80% in *R*-value). The maximum and minimum values in the population were higher than those in the parents, indicating transgressive inheritance. The absolute values of kurtosis and skewness for most traits were <1, except for the kurtosis values of FW and DW were >10.

The ANOVA results revealed significant differences between the year (Y), environment (E), Y × E, and Y × genotype (G) interaction were for all 3 field traits (FER, SH, and NL), and significant genetic effect only existed for FER and NL. The broad-sense heritability of the traits ranged from 14.72 (DW) to 39.69 (FER) (Table 1).

**Table 1.** ANOVA and broad-sense heritability for 8 salt-tolerant traits in RIL population.

Trait	Source	Df	Mean Sq	F value	Pr > F	*h* ^2^_B_ (%)
FER	Y	3	227,788	2,489.581	^c^	39.69
	E	1	2,168	23.697	^c^	
	G	176	383	4.191	^c^	
	Y*E	3	33,134	362.135	^c^	
	Y*G	528	184	2.01	^c^	
	E*G	176	97	1.063		
	Y*E*G	480	88	0.96		
SH	Y	2	3,268	149.608	^c^	26.20
	E	1	47,231	2,161.974	^c^	
	G	176	25	1.137		
	Y*E	1	3,108	142.269	^c^	
	Y*G	176	32	1.462	^c^	
	E*G	176	20	0.894		
	Y*E*G	113	35	1.593	^c^	
NL	Y	2	751.1	1,194.86	^c^	18.42
	E	1	928.8	1,477.521	^c^	
	G	176	0.8	1.243	^a^	
	Y*E	2	182.6	290.532	^c^	
	Y*G	352	0.7	1.193	^a^	
	E*G	176	0.4	0.687		
	Y*E*G	286	0.5	0.835		
GP	E	1	288,349	465.576	^c^	18.00
	G	148	1,196	1.932	^c^	
	E*G	148	459	0.742		
GR	E	1	44,371	89.048	^c^	24.17
	G	148	1,386	2.782	^c^	
	E*G	148	336	0.675		
FW	E	1	9.668	651.928	^c^	34.02
	G	148	0.021	1.428	^b^	
	E*G	148	0.013	0.863		
DW	E	1	0.0019927	9.738	^b^	14.72
	G	148	0.0003959	1.935	^c^	
	E*G	148	0.0001581	0.773		
SL	E	1	33,116	11,025.41	^c^	18.31
	G	148	23	7.683	^c^	
	E*G	148	21	6.844	^c^	

^a,^ ^b,^ ^c^ Significant at P = 0.05, P = 0.01, and P = 0.001, respectively.

FER, field emergence rate; SH, seedling height; NL, number of main stem leaves; GP, germination potential; GR, germination rate; FW, fresh weight; DW, dry weight; GL, germinal length. E, environment (salt stress conditions and normal conditions); G, genotype. *h*^2^_B_, broad-sense heritability.

### Correlation analysis among salt-tolerant related traits

Correlation analysis was performed using the phenotypic values of salt-tolerant traits under E1 and E2, as well as *R*-value (Table 2; Supplementary Table 5). Under E2, GR was significantly positively correlated with FER in 2018 but was no correlated with FER in 2017 and 2019. Under E1, the correlation was not significant between GR and FER in 3 years’ trials (Table 2). NL was significantly positively correlated with SH under E1 and E2 (Supplementary Table 5). No significant correlation was detected for other traits (Supplementary Table 5). Among the 5 traits in 3 datasets (E1, E2, and *R*-value) of the indoor germination test, there was a high positive correlation between GP and GR, as well as FW and DW (Supplementary Table 5).

**Table 2.** Correlation analyses for FER and GR traits under salt stress conditions, normal conditions, and *R*-value.

Environment	Trait	Year	E1						E2					*R*-value			
			GR	FER					GR	FER				GR	FER		
			2018	2017t1	2017t2	2018	2019t1	2019t2	2018	2017t1	2018	2019t1	2019t2	2018	2017t1	2018	2019t1
E1	FER	2017t1	0.006														
		2017t2	0.078	−0.07													
		2018	−0.044	0.048	0.165*a*												
		2019t1	0.131	0.193*a*	0.056	0.099											
		2019t2	0.069	0.188*a*	0.111	0.218*b*	0.036										
E2	GR	2018	0.964^*b*^	−0.002	0.067	−0.036	0.096	0.066									
	FER	2017t1	0.131	0.450^*b*^	−0.105	0.056	0.061	0.256*b*	0.095								
		2018	0.186^*a*^	0.286*b*	0.015	0.254^*b*^	0.17	0.301^*b*^	0.151^*a*^	0.250^*b*^							
		2019t1	0.04	0.13	0.212*b*	0.337*b*	0.225*a*	0.131	0.036	0.183*a*	0.270*b*						
		2019t2	−0.006	0.181*a*	0.086	0.072	0.007	0.642*b*	0.008	0.157*a*	0.249*b*	0.149*a*					
*R*-value	GR	2018	0.682*b*	0.038	0.062	−0.043	0.124	0.029	−0.009	0.162*a*	0.182*a*	0.038	−0.051				
	FER	2017t1	−0.088	0.595*b*	0.006	0.027	0.128	−0.015	−0.066	−0.417*b*	0.082	−0.03	0.062	−0.091			
		2018	−0.175*a*	−0.144	0.106	0.697*b*	−0.045	−0.034	−0.142	−0.109	−0.487*b*	0.088	−0.131	−0.174*a*	−0.034		
		2019t1	0.063	0.122	−0.092	−0.093	0.566*b*	−0.063	0.066	−0.042	−0.035	−0.215*a*	−0.029	−0.02	0.151	−0.061	
		2019t2	0.072	−0.075	−0.013	0.034	−0.038	−0.116	0.056	−0.002	−0.097	−0.104	−0.775*b*	0.065	−0.08	0.123	−0.033

a There was a significant correlation at 0.05 level (bilateral). The critical value for correlation coefficients at probabilities of 0.05 and 0.01 are 0.145 and 0.190, respectively.

b There was a significant correlation at 0.01 level (bilateral).

FER, field emergence rate; GR, germination rate. E1, salt stress conditions; E2, normal conditions; *R*-value, the converted relative index dataset; 2017t1, spring of 2017; 2017t2, summer of 2017; 2019t1, spring of 2019; 2019t2, summer of 2019.

### Single locus QTL analysis

A total of 127 QTL were detected, including 39 for FER, 11 for GP, 12 for GR, 20 for NL, 24 for SH, 4 for FW, 10 for DW, 7 for GL, which explained 1.40–21.03% of PV (Supplementary Table 6). The number of QTL distributed in the A*t* subgenome was similar to that in the D*t* subgenome (72/55). Among these QTL, 47, 52, and 43 QTL were detected in the RIL population under E1, E2, and, *R*-value, respectively.

**Fig. 1.**
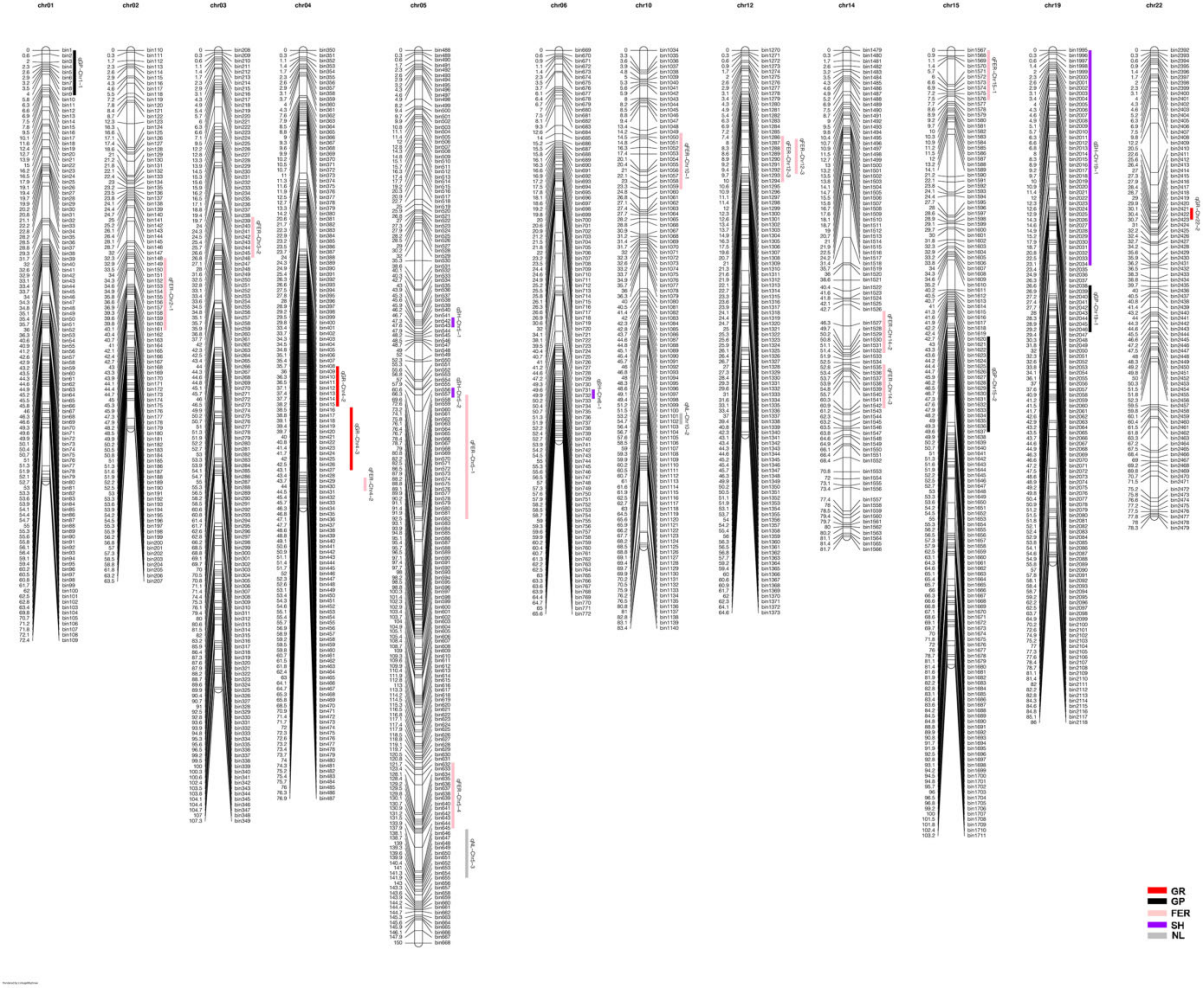
The position of stable or major QTL on the linkage groups. FER, Field emergence rate; GP, germination potential; GR, germination rate; NL, Number of main stem leaves; SH, seedling height. The vertical bars indicate the QTL confidence intervals. Map distances (cM) are shown on the left side of each chromosome.

A total of 22 major QTL (explained PV >10%) or stable QTL (i.e. QTL detected in at least 2 datasets) were detected for 5 traits (FER, GP, GR, NL, and SH) (Table 3 and Fig. 1). A total of 12 stable QTL were detected, of which 2 QTL (*qFER-Chr5-1*, *qFER-Chr10-1*) were detected in E1 and E2, 7 QTL (*qFER-Chr12-3, qFER-Chr15-1, qGP-Chr1-1, qGP-Chr15-2, qGP-Chr19-1, qGR-Chr4-3*, and *qSH-Chr19-1*) were detected in E1 and *R*-value, and 2 QTL (*qFER-Chr5-1, qFER-Chr10-1*) were detected in E2 and *R*-value, respectively. A total of 4 QTL were detected in both E1 and *R*-value at the germination stage with 3 QTL (*qGP-Chr1-1, qGP-Chr15-2*, and *qGP-Chr19-1*) for GP and 1 (*qGR-Chr4-3*) for GR.

**Table 3.** Major QTL or stable QTL for salt-tolerant related traits.

Trait	QTL	Year	Flanking marker		Physical interval		Under E1			Under E2			*R*-value		
			L	R	L	R	LOD	A	Var%	LOD	A	Var%	LOD	A	Var%
**FER (%)**	qFER-Chr2-1*	2017t1	bin159	bin160	17,151,118	18,031,248				3.02	1.48	6.03			
		2017t1	bin161	bin162	27,475,788	24,856,482	2.51	1.21	4.51						
		2019t2	bin166	bin167	40,699,263	NBRI0014				2.62	2.93	4.83			
	***qFER-Chr3-2***	2019t2	bin248	bin249	4,056,547	4,699,558	3.86	−3.36	8.04	8.7	−6.53	17.34			
	qFER-Chr4-2*	2017t1	bin468	bin469	203,840	171,548	3.24	1.93	6.53						
		2018	bin478	bin479	60,980,959	61,232,513	4.7	3.01	8.95						
	*qFER-Chr5-1*	2019t2	bin556	bin557	NAU1042	21,063,321							3.13	11.45	6.67
		2019t2	bin561	bin562	21,454,807	27,411				2.62	−2.92	4.8			
	*qFER-Chr5-4*	2018	bin632	bin633	83,151,316	83,550,175				2.6	−2.32	5.5			
		2018	bin638	bin639	84,715,085	84,316,379	2.8	−2.19	4.71						
	qFER-Chr10-1*	2019t1	bin1052	bin1053	HAU0635	89,548				3.37	2.94	6.94			
		2018	bin1057	bin1058	14,338,049	20,743,965							2.01	4.57	4.04
	*qFER-Chr12-3*	2018	bin1305	bin1306	7,544,817	33,791,740							2.07	4.65	4.2
		2018	bin1311	bin1312	63,877,295	26,826	2.61	2.19	4.72						
	***qFER-Chr14-2***	2017t1	bin1527	bin1528	16,178,195	24,117,598				5.26	−2.09	11.36			
	qFER-Chr14-3*	2018	bin1537	bin1538	30,930,121	42,035,147				2.93	−2.36	5.64			
		2017t1	bin1540	bin1541	45,210,599	54,068,176				3.3	−1.71	7.71			
	*qFER-Chr15-1*	2017t1	bin1571	bin1572	985,874	SWU13909	4.57	−1.71	8.96						
		2017t1	bin1573	bin1574	1,159,622	1,430,078							2.36	−5.51	4.75
**GP (%)**	*qGP-Chr1-1*	2018	bin4	bin4	5,253,419	5,253,419							3.46	3.4	7.98
		2018	bin5	bin6	5,275,311	5,383,055	3.23	2.97	7.68						
	***qGP-Chr15-2***	2018	bin1650	bin1651	37,570,122	37,527,797	2.29	−2.44	5.34				6.97	−6.55	16.95
	***qGP-Chr19-1***	2018	bin2062	bin2063	13,292,260	12,935,942							3.68	4.29	8.52
		2018	bin2063	bin2064	12,935,942	13,782,236	6.5	5.35	16.17						
**GR (%)**	***qGR-Chr4-2***	2018	bin457	bin458	59,365,198	59,380,549							4.73	3.22	10.5
	***qGR-Chr4-3***	2018	bin462	bin463	60,287,107	60,207,332	6.18	4.05	13.67						
		2018	bin467	bin468	193,255	203,840							4.31	3.09	9.62
	***qGR-Chr22-2***	2018	bin2416	bin2417	NAU3966	37,287,704	4.81	−3.53	10.4						
**NL**	qNL-Chr5-3*	2019t2	bin642	bin643	85,089,802	SWU20917				5.25	−0.21	11.22			
		2017t1	bin644	bin645	87,061,817	88,001,753				7.01	−0.2	13.99			
	***qNL-Chr10-2***	2017t1	bin1115	bin1116	NAU3404	SWU20501b	4.77	0.11	10.25						
**SH (cm)**	***qSH-Chr5-1***	2019t1	bin538	bin539	11,142,306	11,079,403	3.72	1.61	11.21						
	***qSH-Chr5-2***	2019t1	bin554	bin555	NAU5330	NAU828	6.45	−2.15	21.03						
	***qSH-Chr6-1***	2019t1	bin750	bin751	66,154,600	77,314,184				6.12	1.31	11.38			
	*qSH-Chr19-1*	2019t2	bin2054	bin2055	8,848,052	8,858,304							2.2	−1.92	4.57
		2019t2	bin2054	bin2055	8,848,052	8,858,304	3.21	−1.02	6.78						

FER, Field emergence rate; GP, germination potential; GR, germination rate; NL, Number of main stem leaves; SH, seedling height; FW, fresh weight; DW, dry weight; GL, germinal length. 2017t1, spring of 2017; 2017t2, summer of 2017; 2019t1, spring of 2019; 2019t2, summer of 2019. E1, salt stress conditions; E2, normal conditions; *R*-value, the converted relative index dataset. A, additive effect. Var%, phenotypic variation explained by a single locus QTL (%). Figures underlined referred to the common QTL detected on 2 datasets on the same year in the present study. QTL noted by “*” referred to common QTL detected on 2 datasets. Bold fonts referred to stable QTL that explained phenotypic variation >10%.

A total of 5 QTL, which explained the PV of 4.04–8.95%, were detected in the field trials at least 2 years with 4 (*qFER-Chr2-1, qFER-Chr4-2, qFER-Chr10-1, qFER-Chr14-3*) for FER and 1 (*qNL-Chr5-3*) for NL, respectively. Four of the stable QTL, *qFER-Chr3-2, qGP-Chr15-2, qGP-Chr19-1*, and *qGR-Chr4-3*, explaining more than 10% of the PV (5.34–17.34%) in at least 1 environment were identified as major QTL. Under E1, the number of QTL received favorable alleles from the female GX1135 (19) was more than that from the male GX100-2 (17). However, the number of QTL received favorable alleles contributed by the female GX1135 (20) was less than that from the male GX100-2 (28) under E2. We also detected many QTL (125) in 1 environment (Supplementary Table 6). These major and stable QTL were used to explore salt tolerance genes in upland cotton.

### Pleiotropic effects

A QTL cluster is defined as a QTL region on a chromosome that contains many QTL associated with different traits (Rong *et al.* 2007). Here, totally, 25 clusters distributed on 16 chromosomes showed pleiotropic effects involving 74 QTL (Table 4; Supplementary Table 7). Among them, 4 clusters were identified on Chr5; 3 were detected on Chr4 and Chr15 each; and 2 were detected on Chr1 and Chr24 each; only 1 was detected on the other 11 chromosomes, respectively.

**Table 4.** Pleiotropic loci for 3 salt-tolerant related traits (FER, GP, and GR).

Cluster	QTL	Year	Flanking marker		Physical interval		Under E1			Under E2			*R*-value		
			L	R	L	R	LOD	A	Var% ^b^	LOD	A	Var%	LOD	A	Var%
Loci-Chr4-1	*qGL-Chr4-1*	2018	bin384	bin385	7,167,948	7,183,670							3.75	3.65	9.59
	*qSH-Chr4-1*	2019t1	bin385	bin386	7,183,670	7,206,099	2.56	1.02	7.50						
	*qFER-Chr4-1*	2018	bin403	bin404	HAU1332	24,618,966							2.89	−5.72	6.41
	*qGR-Chr4-1*	2018	bin439	bin440	SWU21485	57,366,289							2.11	2.20	4.88
Loci-Chr4-2	*qNL-Chr4-1*	2017t1	bin457	bin458	59,365,198	59,380,549	2.09	−0.07	4.34						
	***qGR-Chr4-2***	2018	bin457	bin458	59,365,198	59,380,549							4.73	3.22	10.50
	***qGR-Chr4-3***	2018	bin462	bin463	60,287,107	60,207,332	6.18	4.05	13.67						
		2018	bin467	bin468	193,255	203,840							4.31	3.09	9.62
	*qFER-Chr4-2*	2017t1	bin468	bin469	203,840	171,548	3.24	1.93	6.53						
	*qFER-Chr4-3*	2018	bin478	bin479	60,980,959	61,232,513	4.70	3.01	8.95						
	*qGL-Chr4-2*	2018	bin465	bin466	60,406,140	181,895	3.36	0.41	8.82						
Loci-Chr5-3	*qFER-Chr5-3*	2018	bin611	bin612	52,903,480	54,596,538				4.24	−2.87	8.19			
	*qNL-Chr5-2*	2019t2	bin619	bin620	78,964,923	78,233,305				2.54	0.15	5.23			
	*qGR-Chr5-1*	2018	bin623	bin624	79,979,525	80,357,464	3.11	−2.75	6.53						
Loci-Chr5-4	*qGR-Chr5-2*	2018	bin633	bin634	83,550,175	83,534,339	2.60	−2.51	5.50						
	*qFER-Chr5-4*	2018	bin632	bin633	83,151,316	83,550,175				2.60	−2.32	5.50			
		2018	bin638	bin639	84,715,085	84,316,379	2.80	−2.19	4.71						
	*qDW-Chr5-1*	2018	bin635	bin636	83,593,530	84,429,725	2.93	0.00	8.06						
	*qFW-Chr5-1*	2018	bin638	bin639	84,715,085	84,316,379	2.78	−0.01	7.83						
	*qSH-Chr5-3*	2019t1	bin640	bin641	84,995,410	85,222,043				4.42	−1.13	8.50			
	qNL-Chr5-3*	2019t2	bin642	bin643	85,089,802	SWU20917				5.25	−0.21	11.22			
		2017t1	bin644	bin645	87,061,817	88,001,753				7.01	−0.20	13.99			
Loci-Chr19-1	***qGP-Chr19-1***	2018	bin2062	bin2063	13,292,260	12,935,942							3.68	4.29	8.52
		2018	bin2063	bin2064	12,935,942	13,782,236	6.50	5.35	16.17						
	*qSH-Chr19-2*	2019t2	bin2067	bin2068	19,114,235	19,022,070							3.54	−2.45	7.22
	*qGR-Chr19-1*	2018	bin2069	bin2070	20,025,366	19,397,657				3.76	2.91	8.97			
Loci-Chr2-1	qFER-Chr2-1*	2017t1	bin159	bin160	17,151,118	18,031,248				3.02	1.48	6.03			
		2017t1	bin161	bin162	27,475,788	24,856,482	2.51	1.21	4.51						
		2019t2	bin166	bin167	40,699,263	NBRI0014				2.62	2.93	4.83			
	*qGP-Chr2-1*	2018	bin164	bin165	19,986,238	39,473,418				2.67	−2.28	5.89			
Loci-Chr3-1	*qGP-Chr3-1*	2018	bin298	bin299	79,814,714	82,067,685				3.72	−2.65	8.15			
	*qNL-Chr3-1*	2019t1	bin306	bin307	85,779,151	scaffold725_A03.12291							2.80	3.03	9.08
		2019t1	bin309	bin310	94,235,876	94,283,153							2.21	2.76	7.30
	*qGP-Chr3-2*	2018	bin309	bin310	94,235,876	94,283,153				2.40	−2.21	5.37			
	*qDW-Chr3-1*	2018	bin319	bin320	97,054,248	97,067,325	2.33	0.00	3.23						
	*qFER-Chr3-3*	2019t1	bin347	bin348	99,140,450	NAU2960							2.06	6.84	5.21
Loci-Chr4-3	*qFER-Chr4-2*	2018	bin478	bin479	60,980,959	61,232,513	4.70	3.01	8.95						
	*qGP-Chr4-1*	2018	bin479	bin480	61,232,513	61,282,444				4.23	2.85	9.32			
	*qGL-Chr4-4*	2018	bin484	bin485	61,521,740	61,554,433				3.70	0.76	8.89			
Loci-Chr5-2	*qGP-Chr5-1*	2018	bin596	bin597	32,369,468	32,440,175				2.03	−1.94	4.38			
	*qFER-Chr5-2*	2018	bin600	bin601	34,053,821	33,997,045				2.37	−2.16	4.70			
Loci-Chr15-2	*qGP-Chr15-1*	2018	bin1619	bin1620	10,385,809	10,930,861							2.63	3.97	5.99
	*qFER-Chr15-3*	2018	bin1627	bin1628	NAU874	12,231,379	4.57	3.34	8.44						
Loci-Chr15-3	*qFER-Chr15-4*	2018	bin1647	bin1648	13,202,379	scaffold5226.287	4.37	3.05	8.09						
	***qGP-Chr15-2***	2018	bin1650	bin1651	37,570,122	37,527,797	2.29	−2.44	5.34						
		2018	bin1650	bin1651	37,570,122	37,527,797							6.97	−6.55	16.95

E1, salt stress conditions; E2, normal conditions; *R*-value, the converted relative index dataset; 2017t1, spring of 2017; 2017t2, summer of 2017; 2019t1, spring of 2019; 2019t2, summer of 2019. Var%, phenotypic variation explained by a single locus QTL (%). Figures underlined referred to the common QTL detected on 2 datasets in the same year. QTL noted by “*” referred to common QTL detected on 2 datasets. Bold fonts referred to stable QTL that explained phenotypic variation >10%.

A total of 4 clusters (Loci-Chr4-1, Loci-Chr4-2, Loci-Chr5-3, Loci-Chr5-4) harbor both FER and GR traits. Among them, QTL (*qFER-Chr4-1*) with favorable alleles contributed by the GX100-2 were detected in *R*-value in 2018, and *qGR-Chr4-1* with favorable alleles contributed by the GX1135 detected in *R*-value was involved in Loci-Chr4-1. While the second, Loci-Chr4-2 contains 6 QTL, of which 2 QTL controlled FER (*qFER-Chr4-2*, *qFER-Chr4-3*), which were detected in E1 in 2017t1 and 2018, and 2 controlled GR (*qGR-Chr4-2, qGR-Chr4-3*), which were detected in E1 and *R*-value, and the region also controlled NL (*qNL-Chr4-1*) and GL (*qGL-Chr4-2*). The third, QTL (*qFER-Chr5-3*) controlled FER on Loci-Chr5-3 was detected in E2 in 2018 and QTL *qGR-Chr5-1* that controlled GR detected in E1, which received favorable alleles contributed by the GX100-2. The fourth, 6 QTL were detected on Loci-Chr5-4, of which, *qFER-Chr5-4*, affecting FER was detected in E1and E2 in 2018, and *qGR-Chr5-2* was detected in E1. This cluster also controlled DW, FW, SH, and NL, and the favorable alleles in the QTL originated from the male parent GX100-2.

Only 1 cluster (Loci-Chr19-1) controlled GP and GR in the present study including 1 QTL (*qGP-Chr19-1*) detected in E1 and *R*-value, and another (*qGR-Chr19-1*) detected in E2. The favorable alleles in these 2 QTL were contributed by GX1135. Six clusters (Loci-Chr2-1, Loci-Chr3-1, Loci-Chr4-3, Loci-Chr5-2, Loci-Chr15-2, Loci-Chr15-3) were detected controlling FER and GP.

### Identification of candidate genes in response to salt stress

A total of 4 clusters (Loci-Chr4-1, Loci-Chr4-2, Loci-Chr5-3, Loci-Chr5-4) controlling FER and GR were detected. Two clusters (Loci-Chr4-2 and Loci-Chr5-4) explaining PV of >10% in at least 1 experiment were selected for further analysis. The cluster (Loci-Chr4-2) includes 6 QTL with *qFER-Chr4-2* controlling FER in E1 across 2 years (2017 and 2018), 2 QTL (*qGR-Chr4-2*, *qGR-Chr4-3*) for GR explained 9.63–13.67% of PV in *R*-value and E1, and 3 QTL controlling GP, GL, and NL. The favorable alleles of these QTL were contributed by GX1135 except for *qNL-Chr4-1*. The confidence interval of Loci-Chr4-2 was overlapped between the marker bin 457 and bin 479, which was corresponding to 59,365,198—61,232,513 bp in Chr A04, including 137 genes (*Gh_A04G1024–Gh_A04G1110*). Among them, there are 116 annotated genes and 21 genes with uncharacterized protein information. Loci-Chr5-4 included 6 QTL, of which the QTL *qFER-Chr5-4* controlled FER explained 4.71% (E1) and 5.50% (E2) of PV, respectively, and 1 QTL (*qGR-Chr5-2*) controlled GR explained 5.50% of PV were detected in E1, the loci also controlled NL, SH, FW, and DW. The favorable alleles of these QTL were contributed by GX100-2. The confidence interval of Loci-Chr5-4 was overlapped between the marker bin 633 and bin 645, which was corresponding to 83,534,339–88,001,753 bp in Chr A05. The physical distance of this interval is 4,467,414 bp harboring 175 genes (*Gh_A05G3184*–*Gh_A05G3358*).

We annotated these genes by GO and classified the GO terms. These genes within these 4 clusters were divided into 52 subgroups, belonging to 3 major categories including cellular components (CC), molecular function (MF), and biological processes (BP) (Supplementary Fig. 7 and Supplementary Table 8). GO enrichment of genes within Loci-Chr4-2 was mainly enriched in 3 items: embryo sac central cell differentiation in BP, polar nucleus in CC, and squalene monooxygenase activity in MF (Supplementary Fig. 7, a and b). Genes in Loci-Chr5-4 were mainly enriched in developmentally programmed cell death (BP), senescence-associated vacuole (CC), and phospholipase A2 activity (MF) (Supplementary Fig. 7, c and d).

To better understand the biological function of candidate genes and their metabolic pathways, all genes (70,478) in upland cotton served as background and the candidate genes were annotated and enriched by KEGG (Supplementary Fig. 8). KEGG annotation showed that genes within Loci-Chr4-2 were mainly concentrated in 7 metabolic pathways: carbon metabolism, steroid biosynthesis, sesquiterpenoid, and triterpenoid biosynthesis, phosphatidylinositol signaling system, plant hormone signal transduction, phagosome, and protein processing in the endoplasmic reticulum. It showed that genes within Loci-Chr5-4 were mainly concentrated in 7 metabolic pathways: basal transcription factors, endocytosis, phagosome, plant hormone signal transduction, plant–pathogen interaction, amino sugar, and nucleotide sugar metabolism.

To explore the candidate genes related to salt tolerance, RNA-seq analysis was performed using RNA extracted from the root of GX1135 and GX100-2 at different time points (1, 3, 12, and 48 h) after salt treatment. After differential expression analysis (*P* ≤ 0.01, absolute value fold change ≥2.0) of the 2 parents at different sampling points, 5,131 DEGs were detected (Supplementary Table 9).

**Fig. 2.**
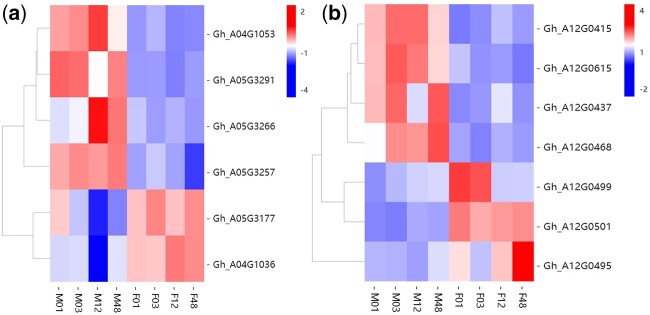
Expression patterns of candidate genes at 1, 3, 12, and 48 h after salt stress in GX1135 and GX100-2. a) Expression patterns of genes in Loci-Chr4-2 and Loci-Chr5-4 differentially expressed in GX1135 and GX100-2 after salt stress. b) Expression patterns of genes in *qFER-Chr12-3* differentially expressed in GX1135 and GX100-2 after salt stress after; M, GX100-2; F, GX1135; 01, 03, 12, and 48 represent 1, 3, 12, and 48 h after salt treatment, respectively.

QTL identified in both salt stress conditions and *R*-value can be used as salt-tolerance-related QTL. Two clusters (Cluster-Chr4-2, Cluster-Chr5-4) controlled 3 or more traits, and 3 QTL (*qFER-Chr12-3, qFER-Chr15-1, qGR-Chr4-3*) detected in at least 2 datasets were selected for further analysis. A total of 370 genes were identified in candidate QTL, of which 334, 32, and 4 genes were identified within *qFER-Chr12-3* (A12, 7,544,817–33,791,740 bp)*, qFER-Chr15-1* (D02, 985,874–1,430,078 bp), *qGR-Chr4-3* (A04, 60,207,332–60,287,107 bp), respectively. We analyzed the expression profiles of all genes within the QTL region and removed non-DEGs. A total of 16 candidate DEGs with opposite expression patterns after salt stress between the 2 parents were screened. Among the candidate genes screened in Loci-Chr4-2 and Loci-Chr5-4, 5 genes (*Gh_A04G1053, Gh_A05G3291, Gh_A05G3266, Gh_A05G3257, Gh_A04G1106*) were upregulated in GX100-2 and downregulated in GX1135 after salt stress treatment, and 4 (*Gh_A04G1046, Gh_A05G3246, Gh_A04G1036, Gh_A05G3177*) showed opposite expression patterns (Fig. 2a; Supplementary Table 10). The confidence interval of *qFER-Chr12-3* was overlapped between marker bin 1305 and bin 1312, corresponding to the reference genome of upland cotton TM-1 7,544,817–33,791,740 bp in Chr A12, the physical distance of this interval is 26,246,923 bp, harboring 334 genes (*Gh_A12G0392–Gh_A12G0725*). We found that 7 DEGs within the QTL *qFER-Chr12-3* presented different expression patterns after 150 mM NaCl treatment. A total of 4 genes (*Gh_A12G0415, Gh_A12G0615, Gh_A12G0437, Gh_A12G0468*) were upregulated in GX100-2 and downregulated at all time points in GX1135 after salt stress treatment, and 3 (*Gh_A12G0499, Gh_A12G0501, Gh_A12G0495*) showed opposite expression patterns after salt stress (Fig. 2b;Supplementary Table 10).

**Fig. 3.**
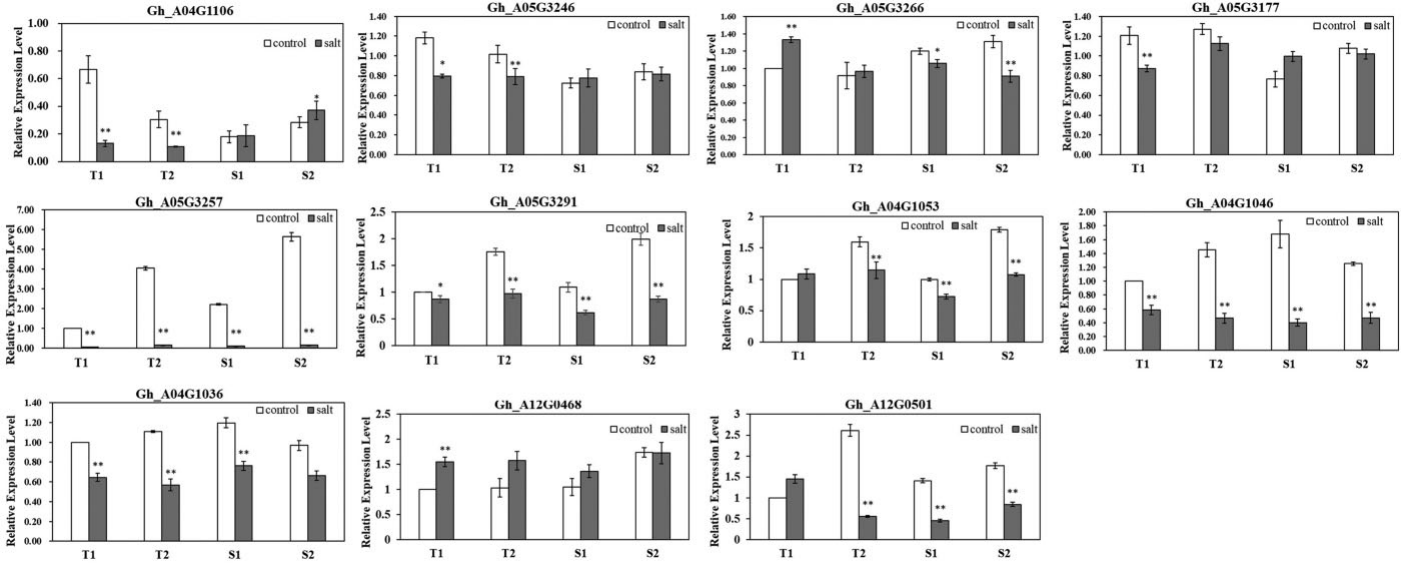
The relative expression levels of candidate genes identified in salt-tolerant lines and salt-sensitive lines using qRT-PCR. The genes’ relative expression levels were determined by 2^−ΔΔCT^ as expressed and were normalized to the expression level of *GhUBQ7* gene. T, salt-tolerant line; S, salt-sensitive line. *^,^ ** significant difference at *P* =* *0.05, 0.01, respectively.

To validate the potential function of candidate genes in salt stress response, the expression patterns of 16 genes were verified with qRT-PCR in 2 salt-tolerance lines (T) and 2 salt-sensitive lines (S) at germination stages after salt stress (Fig. 3). The expression of 5 genes (*Gh_A12G0415, Gh_A12G0615, Gh_A12G0437, Gh_A12G0499, Gh_A12G0495*) were not detectable in salt-tolerance and -sensitive lines. Three genes (*Gh_A04G1106, Gh_A05G3246, Gh_A05G3177*) were significantly downregulated in salt-tolerant lines and no significant difference or upregulated in salt-sensitive lines under salt stress conditions. The gene (*Gh_A05G3266*) encode zinc finger BED domain-containing protein which was downregulated in 2 salt-sensitive lines while upregulated in salt-tolerant lines under salt stress conditions. The other 7 genes showed the same expression pattern in salt-tolerance and salt-sensitive lines after salt stress treatment.

## Discussion

Salt tolerance is a genetically complex trait controlled by many minor-effect genes (Flowers 2004). With the rapid development of SNP arrays and next-generation sequencing technology, the efficiency of QTL mapping applied in cotton salt tolerance was greatly improved. Little research has been reported toward determining the effect of salt stress at the seedling stage in upland cotton, especially under field conditions. In the present study, 5 salt-tolerant related traits (GP, GR, FW, DW, and GL) at germinating stage and 3 (FER, SH, and NL) at seedling stage were evaluated for salt-tolerance, and then QTL mapping was performed to identify genetic loci related to salt tolerance. Then RNA-seq analysis was used to identify genes within the QTL intervals that are responsive to salt stress treatments.

### Comprehensive analysis of salt tolerance at germination and seedling stages

Plants are more sensitive to salt stress at germination and seedling stages than all the other stages (Ahmad *et al.* 2002). Most researches about salt tolerance evaluation were performed in laboratory experiments, such as paper roll systems (Yasir *et al.* 2019), hydroponics (Oluoch *et al.* 2016), and pot experiments (Wang *et al.* 2020) at germination or seedling stage. These methods allow a rapid, accurate, and high-throughput analysis of salt-tolerance at an early growth stage; however, it is difficult to evaluate the actual growth pattern of plants under field salt stress environment. In the present study, an RIL population was used for salt tolerance QTL mapping in both controlled indoor environment and field conditions, which could evaluate the salt tolerance of cotton at germination and seedling stages comprehensively.

Salinity has a strong effect on seed germination, causing reduced GR and affecting seedling morphogenesis in cotton. Previous studies have demonstrated that 150 mM NaCl results in a significant difference in the germination and growth of cotton between the salt-tolerant and salt-sensitive cotton cultivars (Zhang *et al.* 2011; Yasir *et al.* 2019).

It is reported that salt tolerance of the cotton seedlings can be effectively identified at 0.3% NaCl content (3 g/kg), and 2 salt-tolerant traits including the RSR and STL were selected to evaluate the salt tolerance of upland cotton (Sun *et al.* 2018). We found that the total content of water-soluble salt of soil sampling in the saline soil was <3 g/kg in 2017 and 2018, while more than 3 g/kg in 2019 (Guo *et al.* 2021) (Supplementary Table 3). However, identification of cotton salt tolerance in the field is rarely reported. In the field experiment of spring in 2019, FER was <10% in saline soil and the relative FER was the lowest (Supplementary Figs. 1, 3, 4 and Supplementary Table 4). The phenotypic repeatability of field experiments is inevitably affected by changed environments. Salt tolerance of upland cotton could be effectively detected when the concentration of soil salt concentration was 3 g/kg in the field (Guo *et al.* 2021). In the critical period of seedling formation, appropriate field management is critical for preventing the low FER due to the water deficiency. Although the salt concentration in field experiments is difficult to control, we found that repeated experiments in the same plot of saline soil over multiple years can effectively reduce experimental errors.

Salt stress causes both primary and secondary effects in crops. Primary effects include both osmotic and ion-toxicity effects on cells, whereas secondary effects such as oxidative stress, which were damaged to cellular components, and metabolic dysfunction (Zelm *et al.* 2020). In our study, the RIL population and their parents were exposed to salt stress at seedling stage under field saline soil and strictly controlled indoor salt treatment conditions. According to the classification standard of saline soil, the soil sample collected from the saline soil belongs to moderately or highly salinized soil, and the soil sample of normal condition belongs to nonsalinized or lightly salinized soil (Supplementary Table 3). It is shown that there was a significant difference in these salt tolerance traits between salt stress and normal conditions in 3-year trials. Under salt stress conditions, the GP, GR, and FER were significantly decreased in contrast to the control (Supplementary Figs. 4 and 5). The GP, GR, and FER of GX100-2 were higher than that in GX1135 under salt stress conditions, which indicated that GX100-2 was more tolerant to salt stress. It has been reported that salinity was negatively correlated with GP, GR, root length, shoot length, fresh root weight, and fresh shoot weight (Wan *et al.* 2018). Many traits related to salt tolerance are used for QTL mapping in cotton, such as GP and GR (Du *et al.* 2016; Diouf *et al.* 2017), RSR (Sun *et al.* 2018), shoot height (Oluoch *et al.* 2016), FW and dry weight of seedlings, MDA (malondialdehyde), EC, chlorophyll content (Diouf *et al.* 2017), fiber quality, yield, and its components (Zhu *et al.* 2020). Therefore, the mining of key genes that control seed germination and seedling establishment under salt stress conditions to elucidate their underlying molecular mechanisms is an urgent and important objective in salt tolerance breeding in cotton.

### Stable QTL underlying salt tolerance traits

The QTL explained PV more than 10% detected in at least 1 environment was defined as a major QTL. Three QTL (*qGP-Chr1-1, qGP-Chr15-2*, *qGP-Chr19-1*) controlled GP, 2 (*qGR-Chr4-3, qGR-Chr22-1*) controlled GR, and 2 (*qFER-Chr12-3*, *qFER-Chr15-1*) controlled FER that were detected in both E1 and *R*-value (Table 3). Three QTL (*qFER-Chr2-1, qFER-Chr3-2, qFER-Chr5-4*) controlled FER were detected in both E1 and E2; 2 QTL (*qFER-Chr5-1*, *qFER-Chr10-1*) controlled FER were detected in E2 and *R*-value (Table 3). One QTL (*qGR-c7*) for GR located on Chr7 in upland cotton has been submitted in the Cotton QTLdb database (http://www.cottonqtldb.org:8081). In the present study, *qFER-Chr7-1* explained 4.86% of PV was detected in *R*-value, while no QTL was detected in the indoor germination experiment. Sun *et al.* (2019) reported that there are 2 SNPs (i02237Gh, i02243Gh) on Chr D01 (Chr14) that were predicted to be stable genetic loci associated with relative GR under salt stress using GWAS analysis. In the present study, 4 QTL controlling FER located on Chr D01. Among them, 3 were detected under normal condition, and 1 QTL (*qFER-Chr14-4*) was detected in *R*-value. The results of these 2 studies are consistent, which indicated that the QTL mapping under salt stress in the field is repeatable. There are 23 SNPs located on Chr A01, A10, D02, D08, D09, D10, and D11 that were significantly associated with the 2 salt-tolerant related traits, RSR and STL detected by GWAS using 713 upland cotton accessions at the seedling stage (Sun *et al.* 2019). In the present study, we found that QTL for FER were detected on these 7 chromosomes, while only 2 QTL (*qGR-Chr22-1, qGR-Chr22-2*) for GR were detected on Chr D09. Oluoch *et al.* (2016) reported that 2 QTL (*qSh-Chr15-1, qSh-Chr24-1*) for SH (shoot height), 1 QTL (*qSfw-Chr15-1*) for SFW (shoot FW), and 3 QTL (*qRfw-Chr9-1*, *qRfw-Chr15-1, qRfw-Chr26-1*) for RFW (root FW) were detected in both salt stress conditions and normal conditions. In the present study, 1 QTL for SH explaining PV of 5.13% was detected under normal conditions (Table 3). This is consistent with previous research that salt stress-related QTL may be aggregated on specific chromosomes although the salt stress treatments were different.

Salt tolerance is a complex quantitative trait that is controlled by many small-effect genes. A QTL cluster is defined as a densely populated QTL region on a chromosome that contains many QTL associated with different traits (Rong *et al.* 2007). In total, 25 clusters distributed on 16 chromosomes showed pleiotropic effects (Table 4; Supplementary Table 7). Germination and seedling stages are more sensitive to salt stress than other stages in cotton development (Foolad and Jones 1993). The GR and FER are critical features to evaluate the seedling quality and viability under salt stress treatment. In the present study, 4 clusters (Loci-Chr4-1, Loci-Chr4-2, Loci-Chr5-3, Loci-Chr5-4) influencing FER and GR were identified (Table 4). Loci-Chr4-2, which was detected in E1 and *R*-value, controlled 5 salt-tolerant related traits. Of which, 1 QTL controlled FER (*qFER-Chr4-2*) was detected on E1 in 2017 and 2018, and 2 QTL controlled GR (*qGR-Chr4-2, qGR-Chr4-3*) were major QTL, of which *qGR-Chr4-2* was detected in *R*-value and *qGR-Chr4-3* was detected in E1, and the cluster also controls GP, GL, and NL. The favorable alleles in QTL included in Loci-Chr4-2 were originated from the female GX1135 except for the QTL (*qNL-Chr4-1*) (Table 4). These QTL might be the main effect QTL controlling the salt tolerance of upland cotton. The marker interval of bin 457–bin 466 may serve as an important target site for marker-assisted selection in improving salt tolerance.

Previous studies have shown that genes responsible for salt tolerance in the allotetraploid cotton genome were mainly derived from the D*t* subgenome (Oluoch *et al.* 2016; Diouf *et al.* 2017). In the present study, 4 clusters on Chr 5 (Loci-Chr5-1, Loci-Chr5-2, Loci-Chr5-3, and Loci-Chr5-4) affected FER were detected in our study with 2 (Loci-Chr5-3, Loci-Chr5-4) controlling FER and GR, and 1 (Loci-Chr5-2) controlling FER and GP (Table 4). The favorable alleles in QTL included in these 4 clusters on Chr 5 were contributed by the male parent GX100-2 except *qNL-Chr5-2* and *qFER-Chr5-1*. The results suggest that the clusters detected on Chr 5 (A05) may play a positive role in salt stress response.

We mapped a major QTL (*qSH-Chr5-2*) controlling SH located on Chr 5 contributing 21.03% of PV under E1 (Table 3). Similarly, the QTL had been reported as *qPH-chr19-1* of plant height in previous research (Shang *et al.* 2015; Ma *et al.* 2018). The QTL (*qPH-chr19-1*) is linked to the SSR marker NAU5330 (bin 554 in our study), which explained the PV of 4.89–44.9%, 15.17–19.77% for plant height in the present study and the previous one, respectively. It is indicated that the current reported QTL for plant height can be used for plant height improvement.

### Candidate genes involved in salt stress response

Salt tolerance is a complex physiological and biochemical process, which depends on multiple signal transduction pathways and involves a multilevel molecular regulation network, such as the phytohormone-mediated, Ca^2^^+^-dependent, and phosphatidylinositol signals (Chen *et al.* 2021). An increasing number of studies have focused on the roles of transcription factors in response to salt stress in cotton (Yuan *et al.* 2019). In the present study, GX100-2 was more tolerant to salt stress than GX1135 (Supplementary Figs. 4 and 5). The gene *Gh_A05G3246*, encoding calcium-dependent protein kinase (CDPK), was significantly downregulated in salt-tolerant lines after salt treatment, which may act as a negative regulator in salt stress response (Fig. 3). CDPKs have been reported to play important roles in response to salt stress in plants, and overexpressed *CDPK2* in potato promoted ROS scavenging, chlorophyll stability, and the induction of stress-responsive genes conferring tolerance to salinity (Grossi *et al.* 2022). Silencing of 4 CDPKs (*GhCPK8, GhCPK38, GhCPK54*, and *GhCPK55*) severely decreased the basal tolerance to salt stress in cotton (Gao *et al.* 2018). The zinc finger proteins are involved in responsiveness to stress, but the relationship between zinc finger BED domain-containing protein and salt stress has not been reported. In the present study, the gene (*Gh_A05G3266*) encoding zinc finger BED domain-containing protein was increased in salt-sensitive lines after salt treatment.

High concentration of salt stress can cause leaf senescence. The senescence marker genes, SEN4 (senescence 4) and SAG12 (senescence-associated gene 12), were increased rapidly after salt stress treatment (Sakuraba *et al.* 2018). In our study, senescence-related genes (7 *GhSAG39* genes) were enriched within Loci-Chr5-4. These genes may be involved in leaf senescence induced by salt stress. Further expression analysis and gene function studies are needed to validation. Because of limitations related to population type or size, further pinpointing the candidate interval based on the RIL population will be difficult. We plan to perform additional work involving QTL fine mapping and functional verification of salt stress-related genes in future studies.

## Data availability

The frequency distributions of all traits are shown in Supplementary Fig. 1. The locations of QTL are shown in Supplementary Fig. 2. The phenotype of cotton under salt stress condition and normal condition are shown in Supplementary Figs. 3–6. The GO and KEGG annotation of genes within Loci-Chr4-2 and Loci-Chr5-4 are shown in Supplementary Figs. 7 and 8. The RILs used to map QTL in indoor germination study was given in Supplementary Table 1. The total content of water-soluble salt of soil in the field was in Supplementary Table 3. Supplementary Table 4 contains the descriptive statistical analysis for all traits. Supplementary Table 5 contains the correlation analyses for all traits. Supplementary Tables 6 and 7 contain single locus QTL and pleiotropic loci detected in our research, respectively. Supplementary Table 8 contains annotation of genes within Loci-Chr4-2 and Loci-Chr5-4. The FPKM value of DEGs is given in Supplementary Table 9. Supplementary Table 10 contains annotation of candidate salt stress-related genes. All raw sequences and processed data of RNA-seq have been deposited in the NCBI’s GEO database under the accession number GSE186533 (The private link: https://www.ncbi.nlm.nih.gov/geo/query/acc.cgi?acc=GSE186533, enter token azqlyoswlhejvub into the box). The phenotype data and the genotype data used for QTL mapping can be found in Supplementary Tables 11 and 12, respectively. Supplementary Table 13 shows the information of SNP or SSR markers contained in the linkage map.

Supplemental material is available at *G3* online.

## Supplementary Material

jkac099_Figure_S1

jkac099_Figure_S2

jkac099_Figure_S3

jkac099_Figure_S4

jkac099_Figure_S5

jkac099_Figure_S6

jkac099_Figure_S7

jkac099_Figure_S8

jkac099_Table_S1

jkac099_Table_S2

jkac099_Table_S3

jkac099_Table_S4

jkac099_Table_S5

jkac099_Table_S6

jkac099_Table_S7

jkac099_Table_S8

jkac099_Table_S9

jkac099_Table_S10

jkac099_Table_S11

jkac099_Table_S12

jkac099_Table_S13
