# Supplementary material for: Identification of candidate genes involved in salt stress response at germination and seedling stages by QTL mapping in upland cotton
Source: G3 (Bethesda). 2022 Apr 26;12(6):jkac099. doi: 10.1093/g3journal/jkac099 (PMC9157077; doi:10.1093/g3journal/jkac099)
Supplement: jkac099_Figure_S1 [file jkac099_figure_s1.doc]

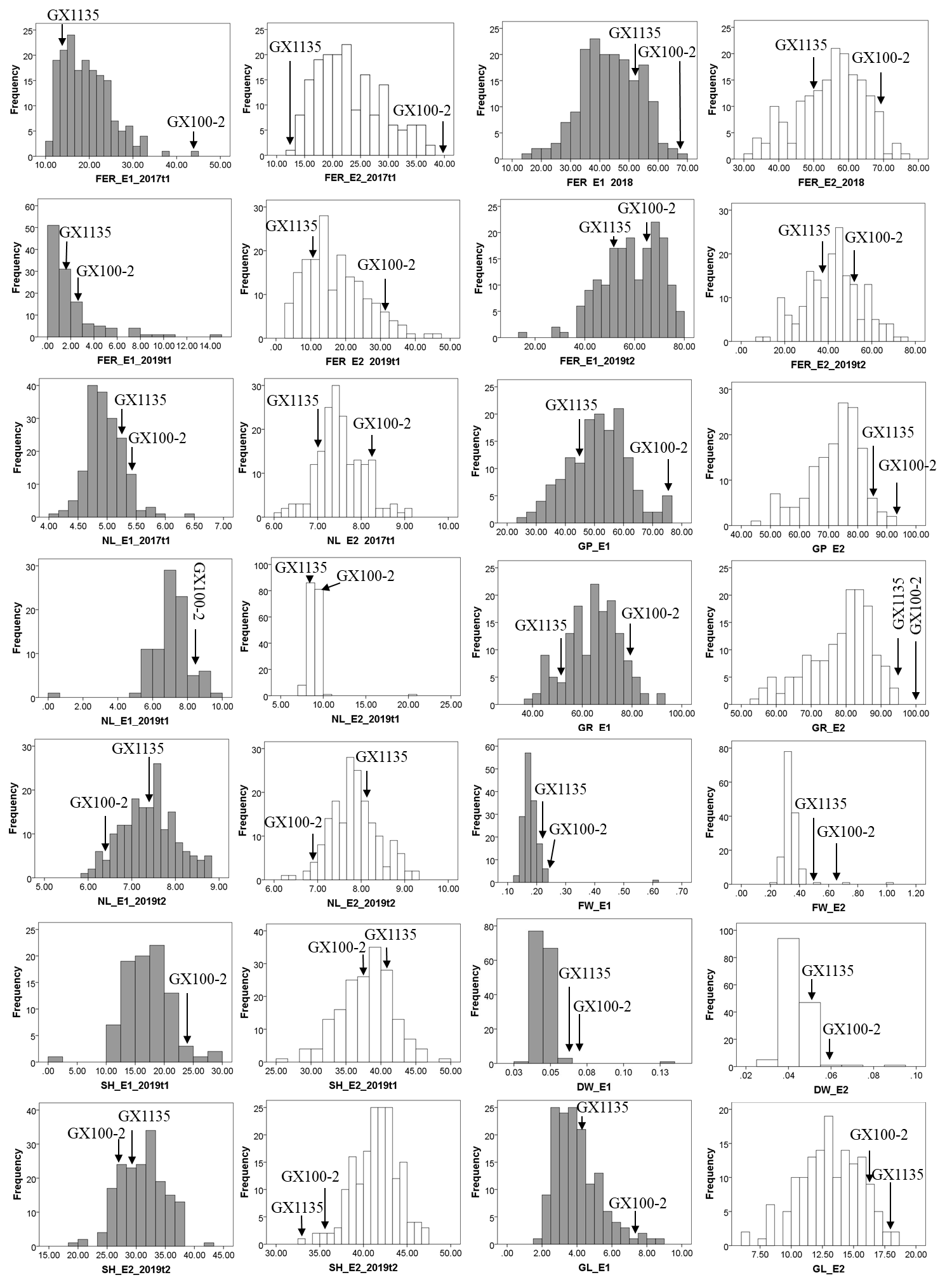


**Figure S1** Frequency distributions of eight salt-tolerant traits in the RILs under salt stress and normal condition in three years. FER, Field emergence rate; NL, Number of main stem leaves; SH, seedling height; GP, germination potential; GR, germination rate; FW, fresh weight; DW, dry weight; GL, germinal length. 2017t1, spring of 2017; 2017t2, summer of 2017; 2019t1, spring of 2019; 2019t2, summer of 2019. E1, salt stress condition; E2, normal condition. Arrows indicate ‘GX1135’ and ‘GX100-2’.
