# Supplementary material for: Identification of candidate genes involved in salt stress response at germination and seedling stages by QTL mapping in upland cotton
Source: G3 (Bethesda). 2022 Apr 26;12(6):jkac099. doi: 10.1093/g3journal/jkac099 (PMC9157077; doi:10.1093/g3journal/jkac099)
Supplement: jkac099_Figure_S2 [file jkac099_figure_s2.doc]

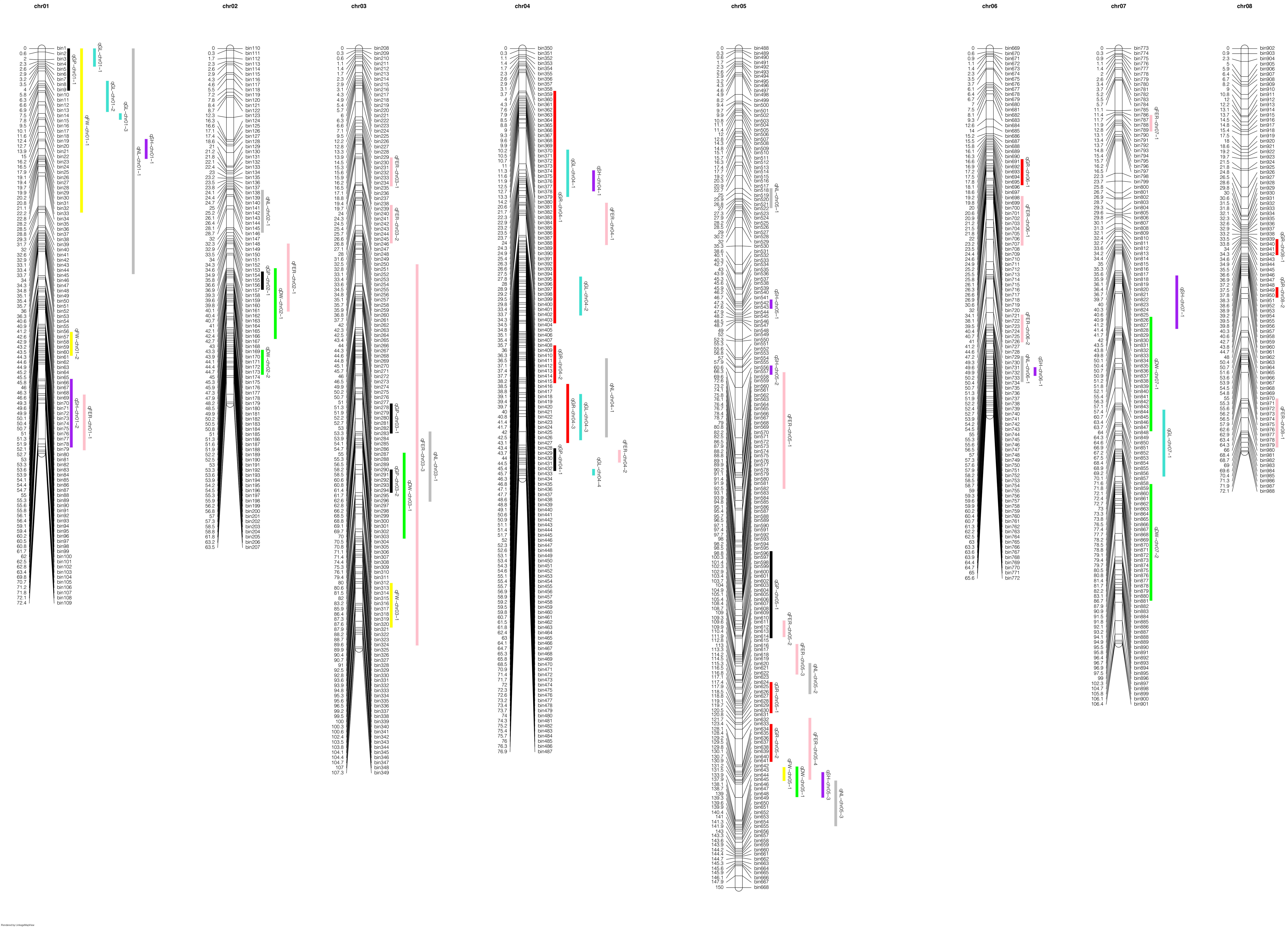


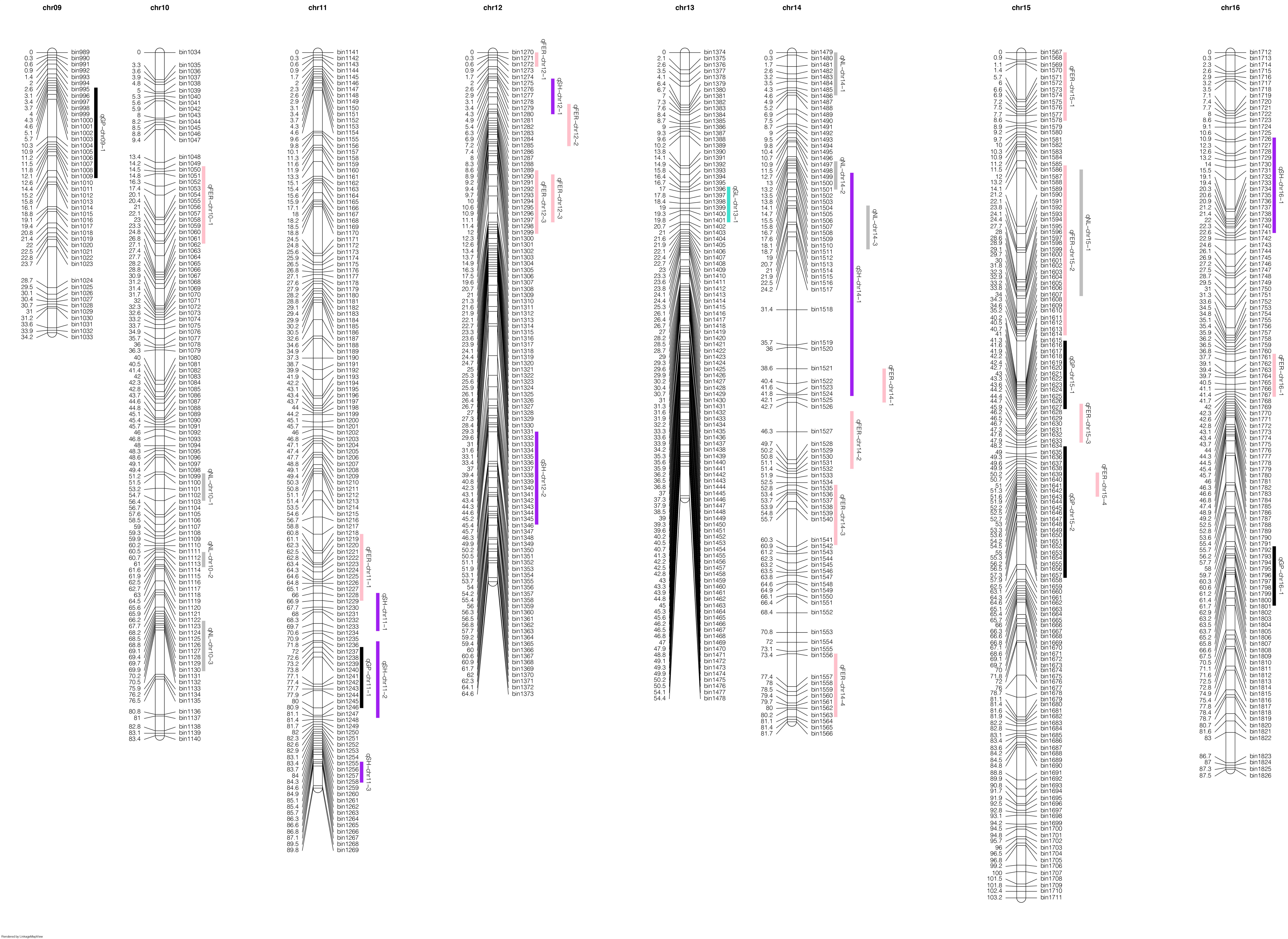


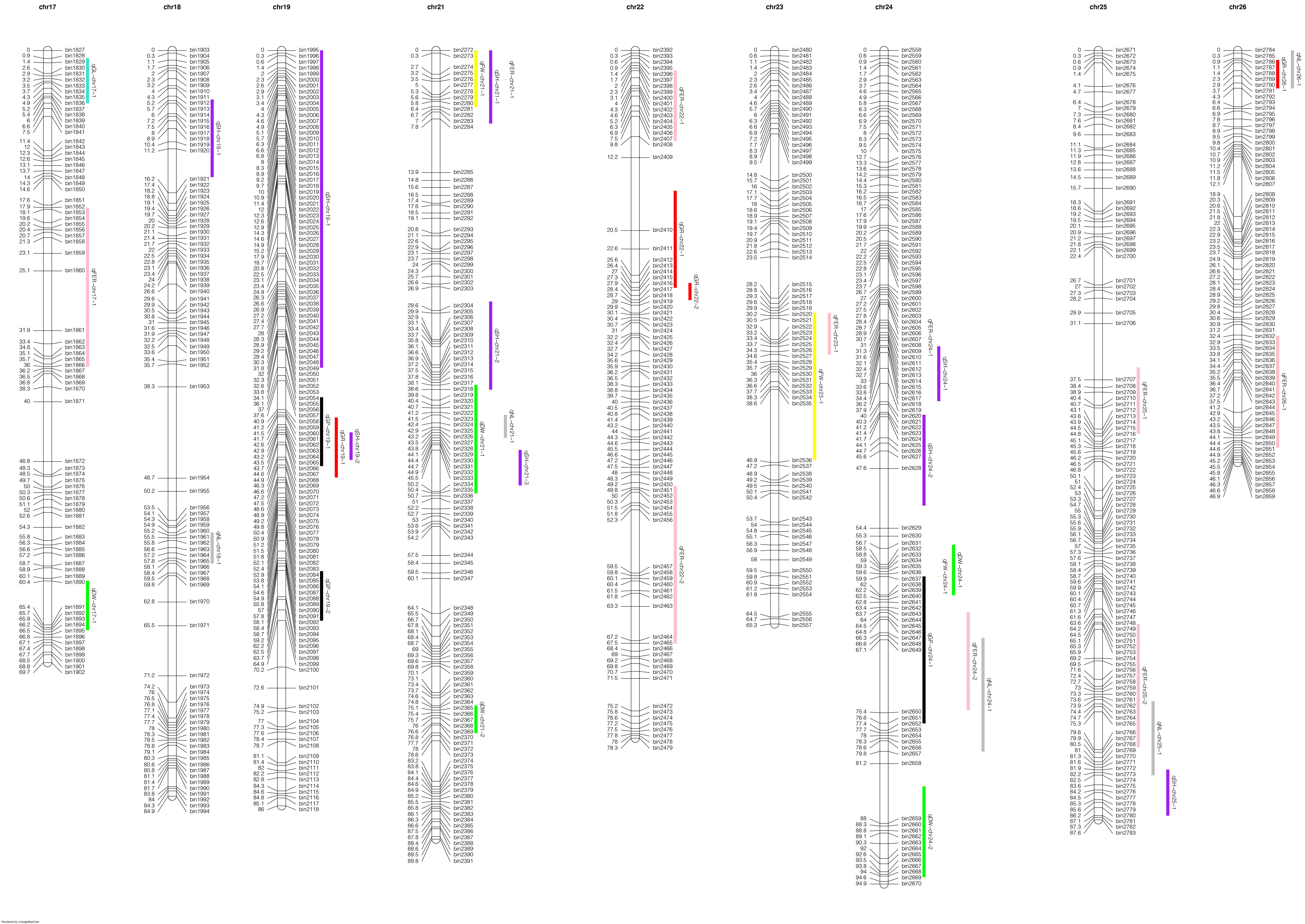


**Figure S2** Locations of QTL controlling salt-tolerant related traits identified in RIL population. FER, Field emergence rate; GP, germination potential; GR, germination rate; NL, Number of main stem leaves; SH, seedling height; FW, fresh weight; DW, dry weight; GL, germinal length. The vertical bars indicate the QTL confidence intervals. Map distances (cM) are shown on the left side of each chromosome.
