# Supplementary material for: Identification of candidate genes involved in salt stress response at germination and seedling stages by QTL mapping in upland cotton
Source: G3 (Bethesda). 2022 Apr 26;12(6):jkac099. doi: 10.1093/g3journal/jkac099 (PMC9157077; doi:10.1093/g3journal/jkac099)
Supplement: jkac099_Figure_S3 [file jkac099_figure_s3.doc]

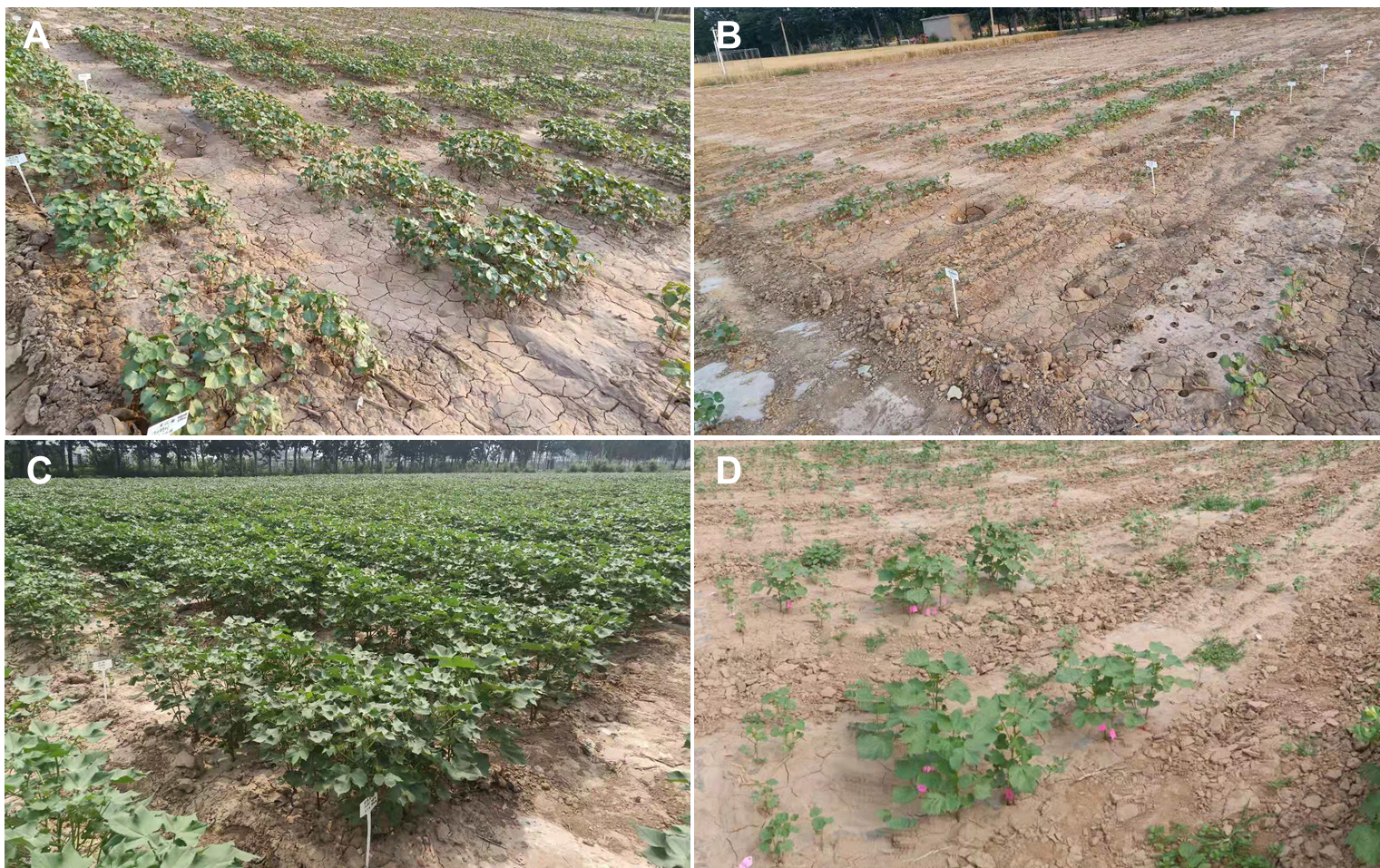


**Figure S3** Performance of cotton in salt stress condition and normal condition (spring of 2019, Quzhou). (A) (C) Performance of cotton in normal condition. (B) (D) Performance of cotton in salt stress condition. Sowing time is from May 6th to May 7th. (A) (B) The shooting time is June 1. (C) (D) The shooting time is June 21st.
