# Supplementary material for: Identification of candidate genes involved in salt stress response at germination and seedling stages by QTL mapping in upland cotton
Source: G3 (Bethesda). 2022 Apr 26;12(6):jkac099. doi: 10.1093/g3journal/jkac099 (PMC9157077; doi:10.1093/g3journal/jkac099)
Supplement: jkac099_Figure_S4 [file jkac099_figure_s4.doc]

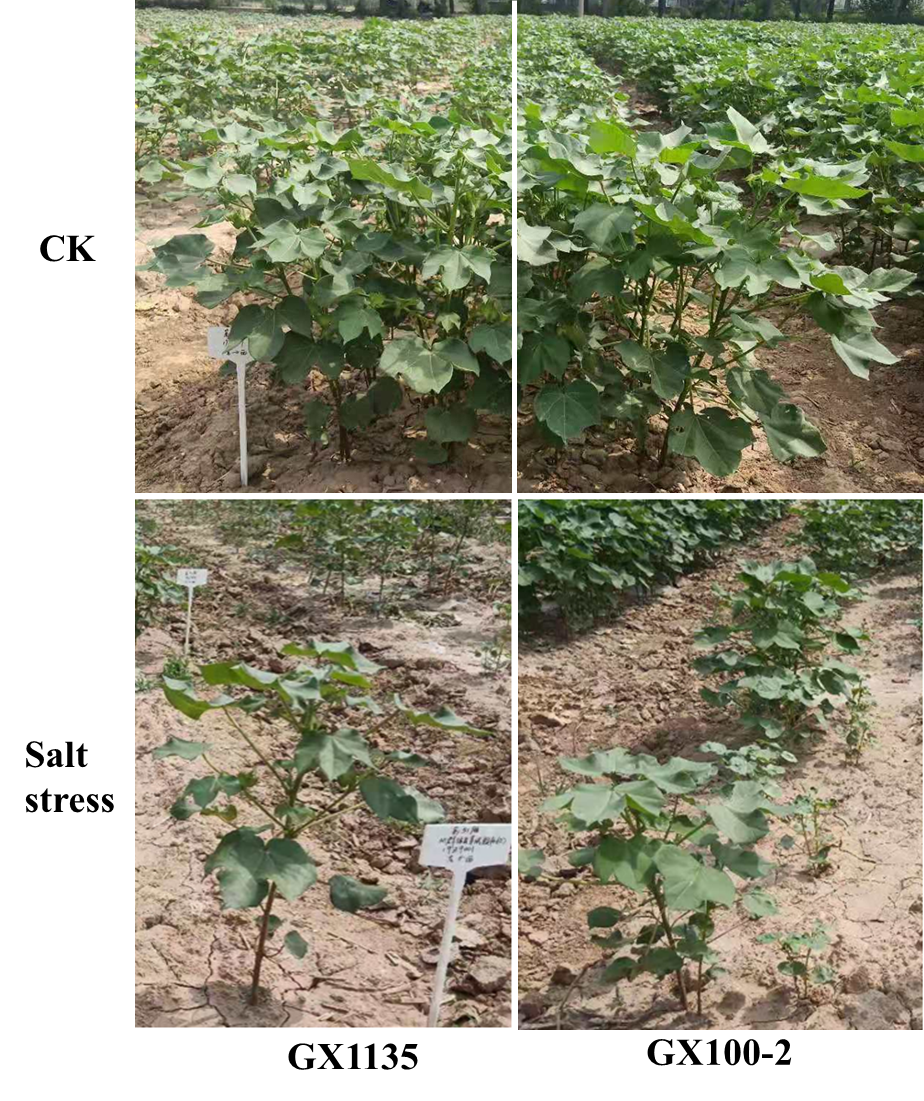


**Figure S4** Performance of GX1135 and GX100-2 seedling in salts stress and normal conditions (June 21^st^, spring of 2019, Quzhou)
