# Supplementary figures and images for: Identification of candidate genes involved in salt stress response at germination and seedling stages by QTL mapping in upland cotton
Source: G3 (Bethesda). 2022 Apr 26;12(6):jkac099. doi: 10.1093/g3journal/jkac099 (PMC9157077; doi:10.1093/g3journal/jkac099)

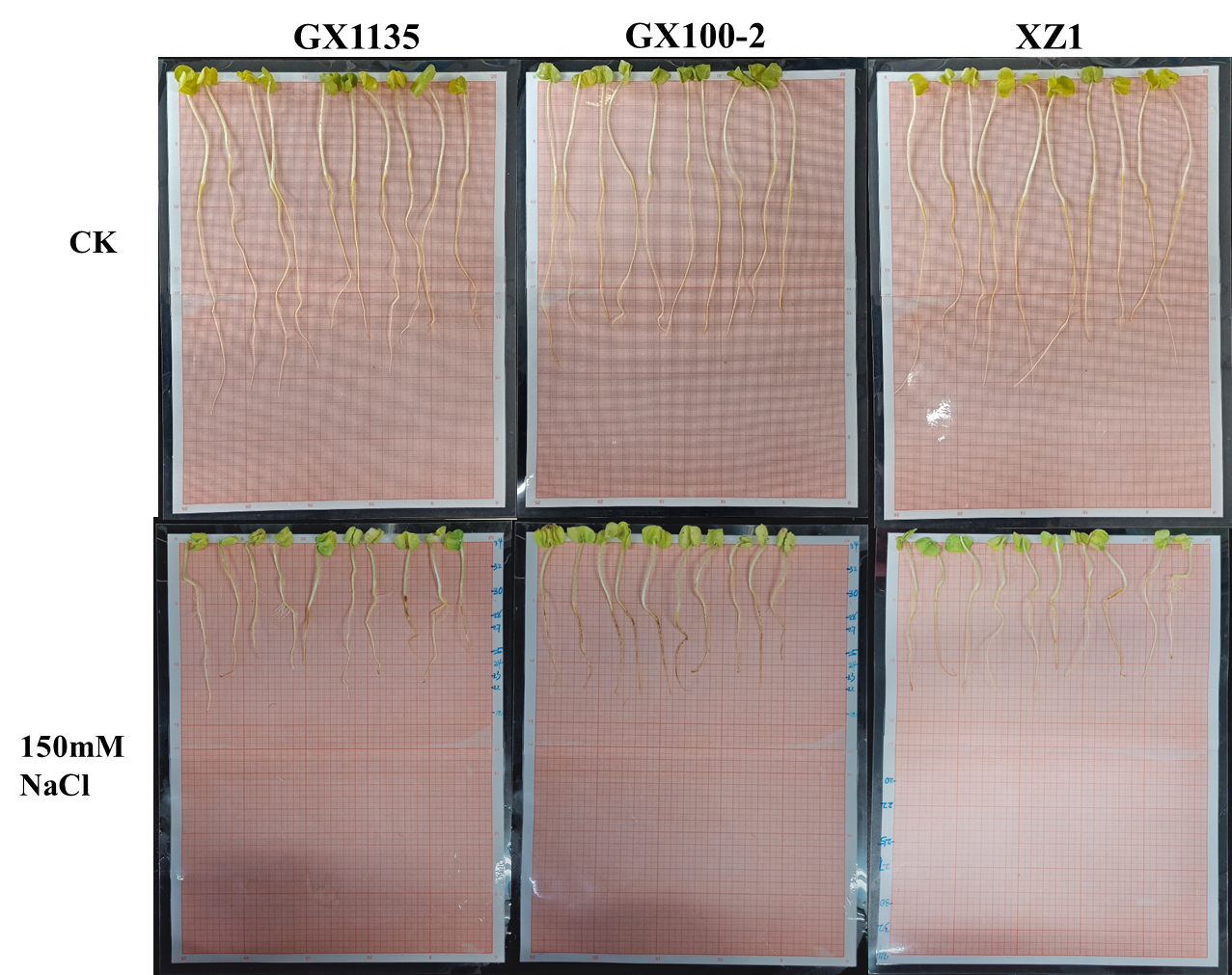


**Figure S6** Performance of GX1135, GX100-2 and XZ1 after salt treatment for seven days

Supplement: jkac099_Figure_S6 [file jkac099_figure_s6.doc]
