# Supplementary material for: Identification of candidate genes involved in salt stress response at germination and seedling stages by QTL mapping in upland cotton
Source: G3 (Bethesda). 2022 Apr 26;12(6):jkac099. doi: 10.1093/g3journal/jkac099 (PMC9157077; doi:10.1093/g3journal/jkac099)
Supplement: jkac099_Figure_S7 [file jkac099_figure_s7.doc]

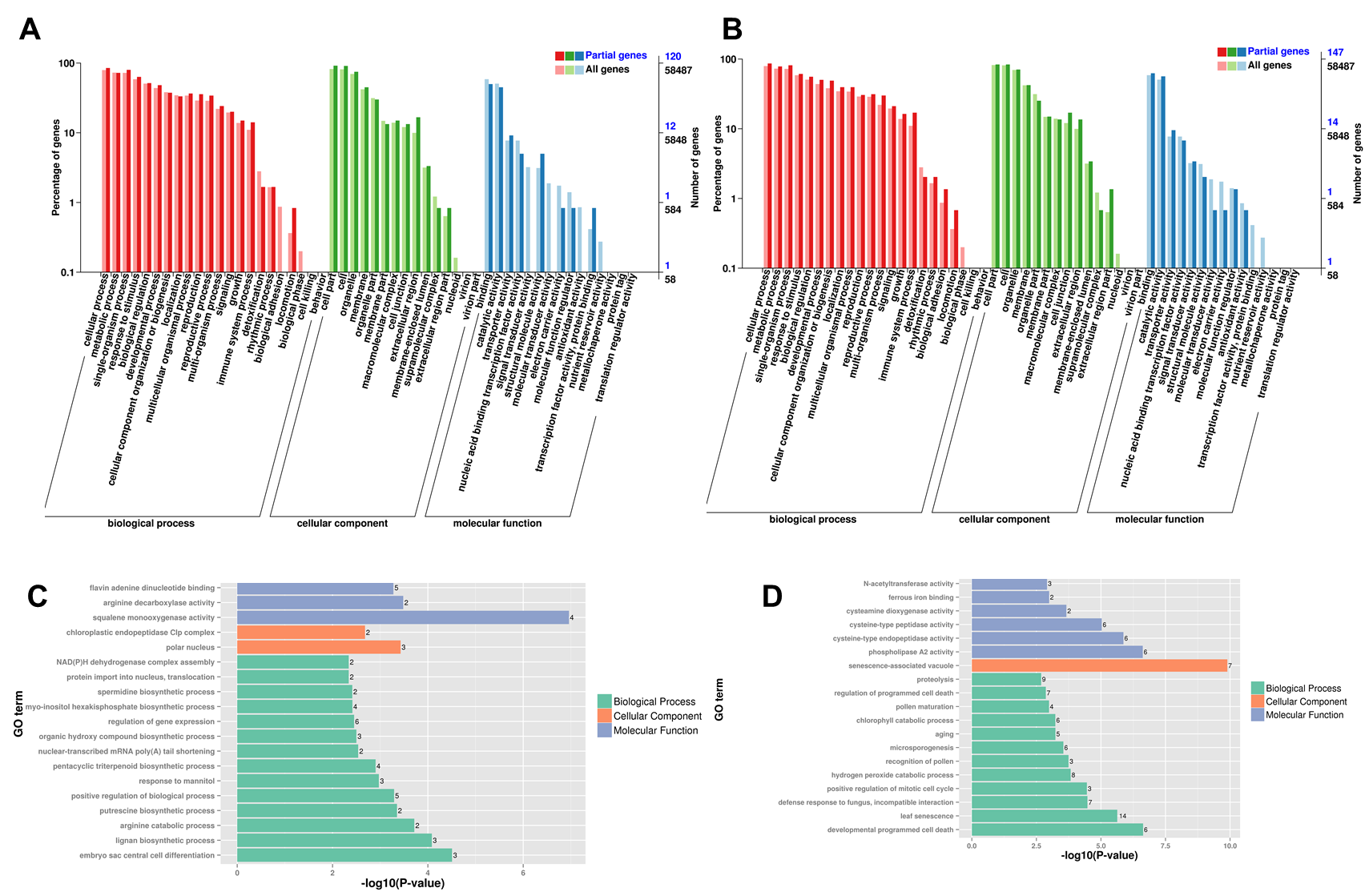


**Figure S7** Gene Ontology (GO) annotation and cluster categories distribution of genes within Loci-Chr4-2 and Loci-Chr5-4. (A) GO classification of candidate genes within Loci-Chr4-2. (B) GO enrichment analysis of candidate genes within Loci-Chr4-2. (C) GO classification of candidate genes within Loci-Chr5-4. (D) GO enrichment analysis of candidate genes within Loci-Chr5-4. The results were assigned to three main categories: biological process, molecular function and cellular component.
