# Supplementary material for: Identification of candidate genes involved in salt stress response at germination and seedling stages by QTL mapping in upland cotton
Source: G3 (Bethesda). 2022 Apr 26;12(6):jkac099. doi: 10.1093/g3journal/jkac099 (PMC9157077; doi:10.1093/g3journal/jkac099)
Supplement: jkac099_Figure_S8 [file jkac099_figure_s8.doc]

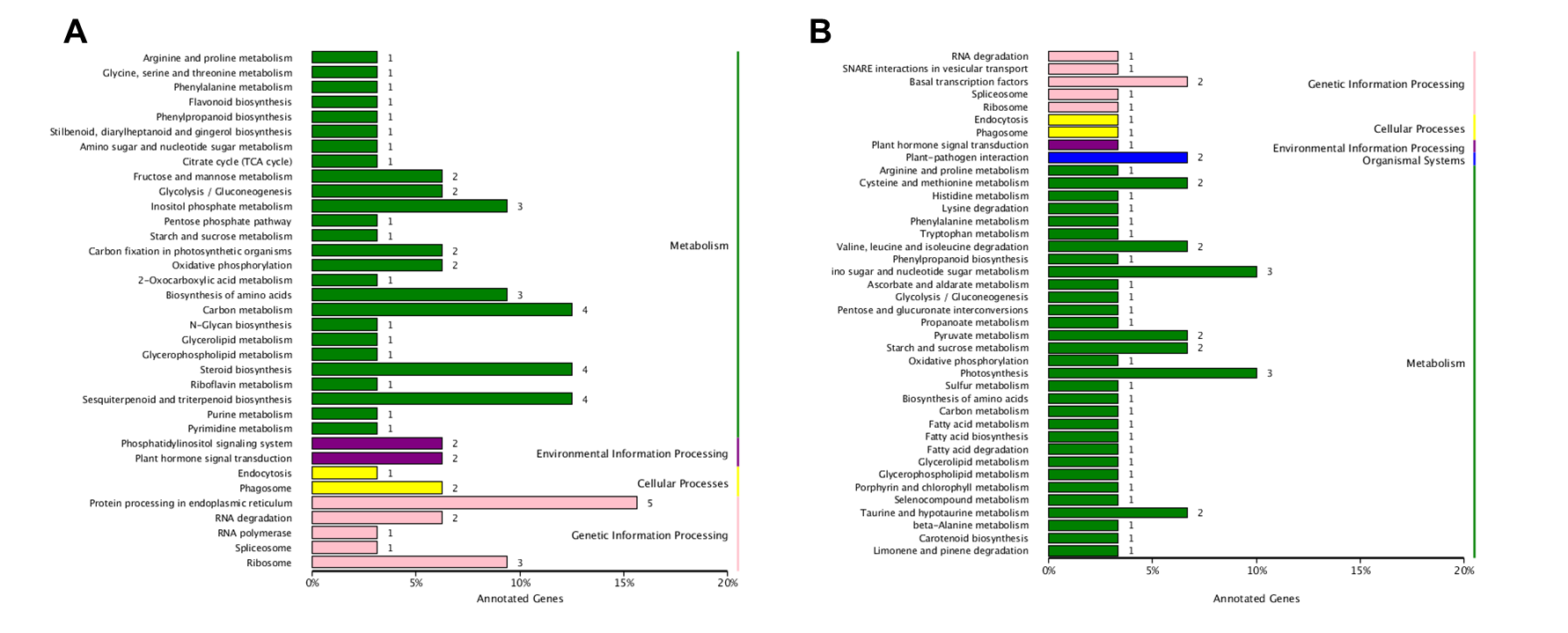


**Figure S8** KEGG classification of genes within Loci-Chr4-2 and Loci-Chr5-4. (A) KEGG classification of candidate genes within Loci-Chr4-2. (B) KEGG functional annotation of candidate genes within Loci-Chr5-4.
