# Supplementary material for: Identification of candidate genes involved in salt stress response at germination and seedling stages by QTL mapping in upland cotton
Source: G3 (Bethesda). 2022 Apr 26;12(6):jkac099. doi: 10.1093/g3journal/jkac099 (PMC9157077; doi:10.1093/g3journal/jkac099)
Supplement: jkac099_Table_S1 [file jkac099_table_s1.doc]

**Table S1 Sample ID of RILs used in indoor germination experiment**

| Sample ID | Sample ID | Sample ID | Sample ID | Sample ID | Sample ID | Sample ID |
| --- | --- | --- | --- | --- | --- | --- |
| RIL003 | RIL030 | RIL056 | RIL081 | RIL109 | RIL133 | RIL160 |
| RIL004 | RIL031 | RIL058 | RIL082 | RIL110 | RIL134 | RIL162 |
| RIL005 | RIL032 | RIL059 | RIL083 | RIL111 | RIL135 | RIL164 |
| RIL006 | RIL033 | RIL060 | RIL085 | RIL112 | RIL137 | RIL166 |
| RIL007 | RIL034 | RIL061 | RIL087 | RIL113 | RIL138 | RIL167 |
| RIL009 | RIL035 | RIL062 | RIL088 | RIL114 | RIL139 | RIL168 |
| RIL010 | RIL036 | RIL063 | RIL089 | RIL115 | RIL140 | RIL170 |
| RIL011 | RIL037 | RIL064 | RIL090 | RIL116 | RIL141 | RIL176 |
| RIL012 | RIL038 | RIL065 | RIL091 | RIL117 | RIL142 | RIL177 |
| RIL013 | RIL039 | RIL066 | RIL092 | RIL118 | RIL143 | RIL178 |
| RIL014 | RIL041 | RIL067 | RIL093 | RIL119 | RIL145 | RIL179 |
| RIL015 | RIL042 | RIL068 | RIL094 | RIL120 | RIL146 |  |
| RIL016 | RIL044 | RIL069 | RIL095 | RIL121 | RIL147 |  |
| RIL017 | RIL045 | RIL070 | RIL096 | RIL122 | RIL148 |  |
| RIL018 | RIL046 | RIL071 | RIL097 | RIL123 | RIL149 |  |
| RIL019 | RIL047 | RIL072 | RIL098 | RIL124 | RIL150 |  |
| RIL020 | RIL048 | RIL074 | RIL099 | RIL125 | RIL151 |  |
| RIL022 | RIL049 | RIL075 | RIL100 | RIL126 | RIL152 |  |
| RIL023 | RIL050 | RIL076 | RIL101 | RIL127 | RIL153 |  |
| RIL025 | RIL051 | RIL077 | RIL104 | RIL128 | RIL155 |  |
| RIL026 | RIL052 | RIL078 | RIL105 | RIL130 | RIL156 |  |
| RIL028 | RIL053 | RIL079 | RIL106 | RIL131 | RIL158 |  |
| RIL029 | RIL054 | RIL080 | RIL107 | RIL132 | RIL159 |  |
