# Supplementary material for: Identification of candidate genes involved in salt stress response at germination and seedling stages by QTL mapping in upland cotton
Source: G3 (Bethesda). 2022 Apr 26;12(6):jkac099. doi: 10.1093/g3journal/jkac099 (PMC9157077; doi:10.1093/g3journal/jkac099)
Supplement: jkac099_Table_S2 [file jkac099_table_s2.doc]

**Table S2** Primer sequences of candidate genes within QTL controlled FER and GR used in qRT-PCR.

| **Gene ID** | **Primer name** | **Sequences (5'-3')** |
| --- | --- | --- |
| *Gh_A04G1039* | A1039-F | ACACCGTGCCTTACCCTTATGC |
|  | A1039-R | ACCAGAACGAAGTTATCCGCCG |
| *Gh_A04G1040* | A1040-F | AGGCACTCCAAGAGCTTGTTCG |
|  | A1040-R | CCCGTAAGCCGAAACGTTAGGT |
| *Gh_A04G1076* | A1076-F | GCAGTGGAAGAACATGCCTCCT |
|  | A1076-R | CCGGCTGCTACGAGATTATTTCCA |
| *Gh_A04G1086* | A1086-F | CGGATAACTTTGCCGGGGAGAA |
|  | A1086-R | CGATGAATTCGTAGCTCCGTGC |
| *Gh_A04G1104* | A1104-F | TGCGCTTGTTCTTCCCTAGACG |
|  | A1104-R | TTTCTGAACCCACCTGACGCTC |
| *Gh_A04G1106* | A1106-F | GCAGTACTAGCCAGGTTGACCG |
|  | A1106-R | ACTTCCAAATTGCCGGACACCA |
| *GhUBQ7* | GhUBQ7-F | GAAGGCATTCCACCTGACCAAC |
|  | GhUBQ7-R | CTTGACCTTCTTCTTCTTGTGCTTG |
