# Supplementary material for: Identification of candidate genes involved in salt stress response at germination and seedling stages by QTL mapping in upland cotton
Source: G3 (Bethesda). 2022 Apr 26;12(6):jkac099. doi: 10.1093/g3journal/jkac099 (PMC9157077; doi:10.1093/g3journal/jkac099)
Supplement: jkac099_Table_S3 [file jkac099_table_s3.doc]

**Table S3** Electrical conductivity and total content of water-soluble salt of soil sampling in the field experiment

| **Sampling Time** | **Soil depth** | **Normal condition** | | **Salt condition** | |
| --- | --- | --- | --- | --- | --- |
|  |  | **EC (μs/cm)** | **ρ (g/kg)** | **EC (μs/cm)** | **ρ (g/kg)** |
| 2017t1 | 0-40 cm | 388.75 | 1.14 | 745.38 | 2.18 |
|  | 0-20 cm | 350.08 | 1.14 | 546.83 | 1.56 |
|  | 20-40 cm | 427.42 | 1.15 | 943.92 | 2.79 |
| 2018t1 | 0-40 cm | 377.28 | 1.33 | 652.50 | 1.87 |
|  | 0-20 cm | 455.33 | 1.67 | 648.22 | 2.08 |
|  | 20-40 cm | 299.22 | 0.98 | 656.78 | 1.66 |
| 2018t2 | 0-40 cm | 371.19 | 1.45 | 511.72 | 1.65 |
|  | 0-20 cm | 313.14 | 1.26 | 323.11 | 1.26 |
|  | 20-40 cm | 416.33 | 1.70 | 700.33 | 2.03 |
| 2019t1 | 0-40 cm | 202.78 | 0.77 | 1130.39 | 3.45 |
|  | 0-20 cm | 197.00 | 0.88 | 1293.22 | 3.97 |
|  | 20-40 cm | 208.56 | 0.65 | 967.56 | 2.93 |
| 2019t2 | 0-40 cm | 203.50 | 0.91 | 1210.83 | 3.79 |
|  | 0-20 cm | 219.67 | 0.95 | 1401.89 | 4.37 |
|  | 20-40 cm | 187.33 | 0.87 | 1019.78 | 3.21 |

2017t1, October 21st, 2017; 2018t1, May 19th, 2018; 2018t2, September 24th, 2019; 2019t1, spring of 2019 (May 16th, 2019); 2019t2, summer of 2019 (July 22nd, 2019). EC, Electrical conductivity; ρ, Total content of water-soluble salt. Data from 2017 to 2018 had been published (Guo *et al.* 2021).
