# Supplementary material for: Identification of candidate genes involved in salt stress response at germination and seedling stages by QTL mapping in upland cotton
Source: G3 (Bethesda). 2022 Apr 26;12(6):jkac099. doi: 10.1093/g3journal/jkac099 (PMC9157077; doi:10.1093/g3journal/jkac099)
Supplement: jkac099_Table_S4 [file jkac099_table_s4.doc]

**Table S4** Descriptive statistical analysis for eight salt-tolerant related traits under salt stress and normal conditions

| **Trait** | **Environment** | **Year** | **RIL population** | | | | | | | **Parent** | |  | **F_1_** | **Control** |
| --- | --- | --- | --- | --- | --- | --- | --- | --- | --- | --- | --- | --- | --- | --- |
|  |  |  | **Mean** | **SD** | **CV%** | **Min** | **Max** | **Skewness** | **Kurtosis** | **GX1135** | **GX100-2** | **significance** | **XZ1** | **RZ816** |
| **FER (%)** | E1 | 2017t1 | 19.64 | 5.61 | 28.58 | 11.36 | 43.75 | 0.93 | 1.36 | 13.64 | 40.3 | ns | 52.84 | 36.93 |
|  | E1 | 2017t2 | 35.83 | 10.3 | 28.76 | 4 | 58 | -0.3 | -0.06 | 46.67 | 40 | - | - | 78 |
|  | E1 | 2018 | 43.42 | 9.98 | 22.99 | 15.87 | 68.27 | -0.14 | -0.21 | 50.72 | 54.81 | ns | 47.12 | 55.29 |
|  | E1 | 2019t1 | 2.12 | 2.39 | 112.78 | 0.28 | 14.77 | 2.44 | 7.52 | 0.57 | 2.84 | ns | 1.14 | 9.38 |
|  | E1 | 2019t2 | 58.8 | 11.68 | 19.87 | 16.48 | 77.84 | -0.53 | -0.01 | 53.13 | 64.2 | ns | 67.05 | 62.78 |
|  | E2 | 2017t1 | 23.21 | 5.93 | 25.55 | 12.5 | 38.07 | 0.52 | -0.51 | 14.77 | 40.91 | ** | 30.68 | 27.27 |
|  | E2 | 2018 | 54.91 | 9.68 | 17.63 | 32.45 | 75 | -0.34 | -0.49 | 50 | 67.79 | * | 73.32 | 77.88 |
|  | E2 | 2019t1 | 17.13 | 8.7 | 50.78 | 2.84 | 46.59 | 0.66 | 0.12 | 9.94 | 30.4 | ns | 46.02 | 31.53 |
|  | E2 | 2019t2 | 42.17 | 13.14 | 31.16 | 7.95 | 74.43 | -0.06 | -0.29 | 37.78 | 52.27 | ns | 36.93 | 5.97 |
|  | R-value | 2017t1 | 87.28 | 24.91 | 28.54 | 45.65 | 197.44 | 1.25 | 2.9 | 92.31 | 98.51 | - | 172.22 | 135.42 |
|  | R-value | 2018 | 81 | 22.57 | 27.87 | 32.67 | 166.67 | 0.99 | 1.53 | 101.44 | 80.85 | - | 64.26 | 70.99 |
|  | R-value | 2019t1 | 15.11 | 29.14 | 192.8 | 0.79 | 300 | 8.06 | 77.25 | 5.71 | 9.35 | - | 2.47 | 29.73 |
|  | R-value | 2019t2 | 149.89 | 43.45 | 28.99 | 81.7 | 302.56 | 1.26 | 1.75 | 140.6 | 122.83 | - | 181.54 | 1052.38 |
| **GP (%)** | E1 | 2018 | 51.29 | 10.29 | 20.06 | 26.57 | 76.17 | -0.06 | -0.16 | 46.05 | 76.53 | ** | 61.98 | 65.6 |
|  | E2 | 2018 | 72.58 | 9.19 | 12.67 | 46.54 | 93.08 | -0.64 | 0.22 | 86.37 | 97.2 | ** | 82.57 | 59.68 |
|  | R-value | 2018 | 70.76 | 11.72 | 16.56 | 39.2 | 96.62 | 0.02 | -0.41 | 53.32 | 78.74 | - | 75.07 | 109.91 |
| **GR (%)** | E1 | 2018 | 64.54 | 10.61 | 16.44 | 38.6 | 92.67 | -0.2 | -0.19 | 52.5 | 84.22 | ** | 87.35 | - |
|  | E2 | 2018 | 78.24 | 9.2 | 11.76 | 53 | 94.8 | -0.7 | -0.13 | 96.42 | 100 | ** | 97.33 | 78.23 |
|  | R-value | 2018 | 82.5 | 9.64 | 11.68 | 55.2 | 108.29 | -0.11 | 0.39 | 54.45 | 84.22 | - | 89.75 | - |
| **NL** | E1 | 2017t1 | 4.98 | 0.32 | 6.46 | 4 | 6.5 | 0.76 | 2.78 | 5.25 | 5.45 | ns | 5.42 | 5.61 |
|  | E1 | 2019t1 | 7.26 | 0.91 | 12.48 | 5 | 9.5 | 0.16 | -0.16 | - | 8.6 | - | 8 | 7.4 |
|  | E1 | 2019t2 | 7.39 | 0.63 | 8.51 | 5.94 | 8.81 | 0.11 | -0.39 | 7.44 | 6.44 | ns | 7.06 | 8.63 |
|  | E2 | 2017t1 | 7.53 | 0.54 | 7.16 | 6.01 | 9.07 | 0.24 | 0.56 | 7.02 | 8.22 | * | 7.51 | 7.74 |
|  | E2 | 2019t1 | 8.97 | 1.04 | 11.64 | 7.07 | 20.7 | 8.02 | 91 | 8.87 | 9.2 | ** | 9.25 | 9.2 |
|  | E2 | 2019t2 | 7.87 | 0.55 | 7.01 | 6.25 | 9.31 | 0.16 | 0.13 | 8 | 6.94 | * | 8.88 | 8.94 |
|  | R-value | 2017t1 | 66.47 | 6.03 | 9.07 | 47.2 | 94.35 | 0.61 | 2.54 | 74.77 | 66.31 | - | 72.16 | 72.44 |
|  | R-value | 2019t1 | 39.49 | 40.87 | 103.48 | 0 | 108.65 | 0.12 | -1.89 | - | 93.48 | - | 86.49 | 80.43 |
|  | R-value | 2019t2 | 94.32 | 9.12 | 9.66 | 75.36 | 121.55 | 0.1 | -0.2 | 92.97 | 92.79 | - | 79.58 | 96.5 |
| **SH (cm)** | E1 | 2019t1 | 17.16 | 3.66 | 21.31 | 11 | 28.8 | 0.8 | 0.75 | - | 23.6 | - | 20 | 18.9 |
|  | E1 | 2019t2 | 31.01 | 3.83 | 12.33 | 19.75 | 42.5 | -0.12 | -0.12 | 29.88 | 27.81 | ns | 31.13 | 43.5 |
|  | E2 | 2019t1 | 37.92 | 3.84 | 10.13 | 25.57 | 48.6 | -0.22 | 0.21 | 41.81 | 38.3 | ** | 43.1 | 48.25 |
|  | E2 | 2019t2 | 41.46 | 2.58 | 6.23 | 33.31 | 47.13 | -0.37 | 0.25 | 32.81 | 35.19 | ** | 43.88 | 48.5 |
|  | R-value | 2019t1 | 44.02 | 10.22 | 23.21 | 0 | 77.01 | -0.13 | 4.13 | - | 61.62 | - | 46.4 | 39.17 |
|  | R-value | 2019t2 | 74.91 | 8.92 | 11.9 | 48.54 | 98.91 | -0.22 | 0.26 | 91.05 | 79.04 | - | 70.94 | 89.69 |
| **FW (g)** | E1 | 2018 | 0.18 | 0.04 | 23.71 | 0.13 | 0.61 | 7.87 | 81.65 | 0.2 | 0.25 | ** | 0.2 | - |
|  | E2 | 2018 | 0.34 | 0.07 | 21.59 | 0.22 | 1 | 5.81 | 46.51 | 0.48 | 0.65 | * | 0.55 | 0.63 |
|  | R-value | 2018 | 51.97 | 11.29 | 21.72 | 23.36 | 164.79 | 6.72 | 67.88 | 41.12 | 38.57 | - | 37.18 | - |
| **DW (g)** | E1 | 2018 | 0.05 | 0.01 | 19.57 | 0.03 | 0.13 | 5.89 | 54.87 | 0.06 | 0.07 | ** | 0.05 | - |
|  | E2 | 2018 | 0.04 | 0.01 | 16.02 | 0.03 | 0.09 | 2.44 | 13.39 | 0.05 | 0.06 | ** | 0.05 | 0.07 |
|  | R-value | 2018 | 106.27 | 16.42 | 15.45 | 51.4 | 268.99 | 6.5 | 66.34 | 122.5 | 122.85 | - | 112.55 | - |
| **GL (cm)** | E1 | 2018 | 4.03 | 1.32 | 32.76 | 1.69 | 8.65 | 1.1 | 1.33 | 4.36 | 7.2 | ** | 6.65 | - |
|  | E2 | 2018 | 13.12 | 2.46 | 18.74 | 6.59 | 18.11 | -0.3 | -0.28 | 17.64 | 16.05 | ns | 28.67 | 23.64 |
|  | R-value | 2018 | 31.74 | 11.21 | 35.33 | 14.07 | 73.99 | 1.02 | 1.25 | 15.94 | 27.63 | - | 23.18 | - |

FER, Field emergence rate; GP, germination potential; GR, germination rate; SH, seedling height; NL, Number of main stem leaves; FW, fresh weight; DW, dry weight; GL, germinal length. 2017t1, spring of 2017; 2017t2, summer of 2017; 2019t1, spring of 2019; 2019t2, summer of 2019. E1, salt stress condition; E2, normal condition, R-value, relative index dataset. SD, Mean values ± standard deviation values. CV (%), Coefficient of variation (%); *, ** indicate significant difference between GX1135 and GX100-2 at P=0.05 and P=0.01 respectively using students t test; ns means not significant; -, data missing.
