# Supplementary material for: Identification of candidate genes involved in salt stress response at germination and seedling stages by QTL mapping in upland cotton
Source: G3 (Bethesda). 2022 Apr 26;12(6):jkac099. doi: 10.1093/g3journal/jkac099 (PMC9157077; doi:10.1093/g3journal/jkac099)
Supplement: jkac099_Table_S6 [file jkac099_table_s6.doc]

**Table S6 Single locus QTLs for eight salt-tolerant related traits in salt stress condition, normal condition and R-value**

| **Trait** | **QTL** | **Year** | **Flanking marker** | | **Under E1** | | | **Under E2** | | | **R-value** | | |
| --- | --- | --- | --- | --- | --- | --- | --- | --- | --- | --- | --- | --- | --- |
|  |  |  | **L** | **R** | **LOD** | **A** | **Var%** | **LOD** | **A** | **Var%** | **LOD** | **A** | **Var%** |
| **FER** | *qFER-Chr1-1* | 2019t2 | bin105 | bin106 | 3.02 | 3.09 | 6.21 |  |  |  |  |  |  |
|  | *qFER-Chr2-1** | 2017t1 | bin159 | bin160 |  |  |  | 3.02 | 1.48 | 6.03 |  |  |  |
|  |  | 2017t1 | bin161 | bin162 | 2.51 | 1.21 | 4.51 |  |  |  |  |  |  |
|  |  | 2019t2 | bin166 | bin167 |  |  |  | 2.62 | 2.93 | 4.83 |  |  |  |
|  | *qFER-Chr3-1* | 2019t2 | bin241 | bin242 |  |  |  | 2.62 | -3.35 | 5.65 |  |  |  |
|  | ***qFER-Chr3-2*** | 2019t2 | bin248 | bin249 | 3.86 | -3.36 | 8.04 | 8.70 | -6.53 | 17.34 |  |  |  |
|  | *qFER-Chr3-3* | 2019t1 | bin347 | bin348 |  |  |  |  |  |  | 2.06 | 6.84 | 5.21 |
|  | *qFER-Chr4-1* | 2018 | bin403 | bin404 |  |  |  |  |  |  | 2.89 | -5.72 | 6.41 |
|  | *qFER-Chr4-2** | 2017t1 | bin468 | bin469 | 3.24 | 1.93 | 6.53 |  |  |  |  |  |  |
|  |  | 2018 | bin478 | bin479 | 4.70 | 3.01 | 8.95 |  |  |  |  |  |  |
|  | *qFER-Chr5-1* | 2019t2 | bin556 | bin557 |  |  |  |  |  |  | 3.13 | 11.45 | 6.67 |
|  |  | 2019t2 | bin561 | bin562 |  |  |  | 2.62 | -2.92 | 4.80 |  |  |  |
|  | *qFER-Chr5-2* | 2018 | bin600 | bin601 |  |  |  | 2.37 | -2.16 | 4.70 |  |  |  |
|  | *qFER-Chr5-3* | 2018 | bin611 | bin612 |  |  |  | 4.24 | -2.87 | 8.19 |  |  |  |
|  | *qFER-Chr5-4* | 2018 | bin632 | bin633 |  |  |  | 2.60 | -2.32 | 5.50 |  |  |  |
|  |  | 2018 | bin638 | bin639 | 2.80 | -2.19 | 4.71 |  |  |  |  |  |  |
|  | *qFER-Chr6-1* | 2019t2 | bin717 | bin718 |  |  |  |  |  |  | 2.72 | -10.19 | 5.32 |
|  | *qFER-Chr6-2* | 2019t2 | bin734 | bin735 |  |  |  | 4.10 | 3.69 | 7.69 |  |  |  |
|  | *qFER-Chr7-1* | 2018 | bin792 | bin793 |  |  |  |  |  |  | 2.40 | 5.03 | 4.86 |
|  | *qFER-Chr8-1* | 2018 | bin979 | bin980 |  |  |  | 3.06 | 2.42 | 5.90 |  |  |  |
|  | *qFER-Chr10-1** | 2019t1 | bin1052 | bin1053 |  |  |  | 3.37 | 2.94 | 6.94 |  |  |  |
|  |  | 2018 | bin1057 | bin1058 |  |  |  |  |  |  | 2.01 | 4.57 | 4.04 |
|  | *qFER-Chr11-1* | 2019t1 | bin1225 | bin1226 |  |  |  | 2.28 | 1.88 | 4.60 |  |  |  |
|  | *qFER-Chr12-1* | 2017t2 | bin1270 | bin1271 | 2.22 | 2.26 | 4.61 |  |  |  |  |  |  |
|  | *qFER-Chr12-2* | 2018 | bin1295 | bin1296 |  |  |  |  |  |  | 2.35 | 4.98 | 4.75 |
|  | *qFER-Chr12-3* | 2018 | bin1305 | bin1306 |  |  |  |  |  |  | 2.07 | 4.65 | 4.20 |
|  |  | 2018 | bin1311 | bin1312 | 2.61 | 2.19 | 4.72 |  |  |  |  |  |  |
|  | *qFER-Chr14-1* | 2017t1 | bin1523 | bin1524 |  |  |  | 2.93 | -1.51 | 6.19 |  |  |  |
|  | ***qFER-Chr14-2*** | 2017t1 | bin1527 | bin1528 |  |  |  | 5.26 | -2.09 | 11.36 |  |  |  |
|  | *qFER-Chr14-3** | 2018 | bin1537 | bin1538 |  |  |  | 2.93 | -2.36 | 5.64 |  |  |  |
|  |  | 2017t1 | bin1540 | bin1541 |  |  |  | 3.30 | -1.71 | 7.71 |  |  |  |
|  | *qFER-Chr14-4* | 2017t1 | bin1557 | bin1558 |  |  |  |  |  |  | 3.12 | 6.39 | 6.35 |
|  | *qFER-Chr15-1* | 2017t1 | bin1571 | bin1572 | 4.57 | -1.71 | 8.96 |  |  |  |  |  |  |
|  |  | 2017t1 | bin1573 | bin1574 |  |  |  |  |  |  | 2.36 | -5.51 | 4.75 |
|  | *qFER-Chr15-2* | 2018 | bin1594 | bin1595 | 2.36 | -2.25 | 4.48 |  |  |  |  |  |  |
|  | *qFER-Chr15-3* | 2018 | bin1627 | bin1628 | 4.57 | 3.34 | 8.44 |  |  |  |  |  |  |
|  | *qFER-Chr15-4* | 2018 | bin1647 | bin1648 | 4.37 | 3.05 | 8.09 |  |  |  |  |  |  |
|  | *qFER-Chr16-1* | 2019t1 | bin1761 | bin1762 |  |  |  | 4.38 | -2.73 | 9.61 |  |  |  |
|  | *qFER-Chr17-1* | 2017t1 | bin1859 | bin1860 |  |  |  | 2.10 | -1.48 | 4.20 |  |  |  |
|  | *qFER-Chr21-1* | 2019t1 | bin2275 | bin2276 |  |  |  |  |  |  | 1.63 | -6.77 | 5.20 |
|  | *qFER-Chr22-1* | 2018 | bin2403 | bin2404 | 2.73 | -2.23 | 4.93 |  |  |  |  |  |  |
|  | *qFER-Chr22-2* | 2019t2 | bin2457 | bin2458 | 2.78 | -2.84 | 5.70 |  |  |  |  |  |  |
|  | *qFER-Chr23-1* | 2019t2 | bin2524 | bin2525 |  |  |  |  |  |  | 2.28 | -9.41 | 4.44 |
|  | *qFER-Chr24-1* | 2019t1 | bin2613 | bin2614 |  |  |  |  |  |  | 1.61 | -6.16 | 4.04 |
|  | *qFER-Chr24-2* | 2017t2 | bin2644 | bin2645 | 2.41 | 2.38 | 5.00 |  |  |  |  |  |  |
|  | *qFER-Chr25-1* | 2019t2 | bin2709 | bin2710 |  |  |  |  |  |  | 3.27 | 11.36 | 6.56 |
|  | *qFER-Chr25-2* | 2017t1 | bin2753 | bin2754 |  |  |  | 2.10 | 1.23 | 4.15 |  |  |  |
|  | *qFER-Chr26-1* | 2017t1 | bin2840 | bin2841 | 2.22 | 1.21 | 4.51 |  |  |  |  |  |  |
|  |  | 2017t1 | bin2845 | bin2846 | 3.09 | 1.40 | 6.20 |  |  |  |  |  |  |
| **GP** | *qGP-Chr1-1* | 2018 | bin4 | bin4 |  |  |  |  |  |  | 3.46 | 3.40 | 7.98 |
|  |  | 2018 | bin5 | bin6 | 3.23 | 2.97 | 7.68 |  |  |  |  |  |  |
|  | *qGP-Chr2-1* | 2018 | bin164 | bin165 |  |  |  | 2.67 | -2.28 | 5.89 |  |  |  |
|  | *qGP-Chr3-1* | 2018 | bin298 | bin299 |  |  |  | 3.72 | -2.65 | 8.15 |  |  |  |
|  | *qGP-Chr3-2* | 2018 | bin309 | bin310 |  |  |  | 2.40 | -2.21 | 5.37 |  |  |  |
|  | *qGP-Chr4-1* | 2018 | bin479 | bin480 |  |  |  | 4.23 | 2.85 | 9.32 |  |  |  |
|  | *qGP-Chr5-1* | 2018 | bin596 | bin597 |  |  |  | 2.03 | -1.94 | 4.38 |  |  |  |
|  | *qGP-Chr15-1* | 2018 | bin1619 | bin1620 |  |  |  |  |  |  | 2.63 | 3.97 | 5.99 |
|  | ***qGP-Chr15-2*** | 2018 | bin1650 | bin1651 | 2.29 | -2.44 | 5.34 |  |  |  | 6.97 | -6.55 | 16.95 |
|  | *qGP-Chr16-1* | 2018 | bin1805 | bin1806 |  |  |  | 2.09 | 2.01 | 4.19 |  |  |  |
|  | ***qGP-Chr19-1*** | 2018 | bin2062 | bin2063 |  |  |  |  |  |  | 3.68 | 4.29 | 8.52 |
|  |  | 2018 | bin2063 | bin2064 | 6.50 | 5.35 | 16.17 |  |  |  |  |  |  |
|  | *qGP-Chr19-2* | 2018 | bin2096 | bin2097 | 2.25 | -3.05 | 5.27 |  |  |  |  |  |  |
| **GR** | *qGR-Chr4-1* | 2018 | bin439 | bin440 |  |  |  |  |  |  | 2.11 | 2.20 | 4.88 |
|  | ***qGR-Chr4-2*** | 2018 | bin457 | bin458 |  |  |  |  |  |  | 4.73 | 3.22 | 10.50 |
|  | ***qGR-Chr4-3*** | 2018 | bin462 | bin463 | 6.18 | 4.05 | 13.67 |  |  |  |  |  |  |
|  |  | 2018 | bin467 | bin468 |  |  |  |  |  |  | 4.31 | 3.09 | 9.62 |
|  | *qGR-Chr5-1* | 2018 | bin623 | bin624 | 3.11 | -2.75 | 6.53 |  |  |  |  |  |  |
|  | *qGR-Chr5-2* | 2018 | bin633 | bin634 | 2.60 | -2.51 | 5.50 |  |  |  |  |  |  |
|  | *qGR-Chr6-1* | 2018 | bin708 | bin709 | 2.00 | 2.20 | 4.15 |  |  |  |  |  |  |
|  | *qGR-Chr8-1* | 2018 | bin942 | bin943 |  |  |  | 5.02 | 3.38 | 12.23 |  |  |  |
|  | *qGR-Chr8-2* | 2018 | bin960 | bin961 |  |  |  | 2.70 | 2.53 | 6.81 |  |  |  |
|  | *qGR-Chr19-1* | 2018 | bin2069 | bin2070 |  |  |  | 3.76 | 2.91 | 8.97 |  |  |  |
|  | *qGR-Chr22-1* | 2018 | bin2411 | bin2412 | 2.86 | -2.76 | 6.36 |  |  |  | 3.64 | -2.83 | 7.94 |
|  | ***qGR-Chr22-2*** | 2018 | bin2416 | bin2417 | 4.81 | -3.53 | 10.40 |  |  |  |  |  |  |
|  | *qGR-Chr26-1* | 2018 | bin2790 | bin2791 |  |  |  |  |  |  | 3.41 | -2.85 | 8.47 |
| **NL** | *qNL-Chr1-1* | 2019t1 | bin37 | bin38 |  |  |  |  |  |  | 2.04 | 2.55 | 6.44 |
|  | *qNL-Chr2-1* | 2019t1 | bin146 | bin147 |  |  |  | 2.00 | -0.18 | 2.74 |  |  |  |
|  | *qNL-Chr3-1* | 2019t1 | bin306 | bin307 |  |  |  |  |  |  | 2.80 | 3.03 | 9.08 |
|  |  | 2019t1 | bin309 | bin310 |  |  |  |  |  |  | 2.21 | 2.76 | 7.30 |
|  | *qNL-Chr4-1* | 2017t1 | bin457 | bin458 | 2.09 | -0.07 | 4.34 |  |  |  |  |  |  |
|  | *qNL-Chr5-1* | 2017t1 | bin522 | bin523 |  |  |  |  |  |  | 10.90 | 2.37 | 1.75 |
|  | *qNL-Chr5-2* | 2019t2 | bin619 | bin620 |  |  |  | 2.54 | 0.15 | 5.23 |  |  |  |
|  | ***qNL-Chr5-3**** | 2019t2 | bin642 | bin643 |  |  |  | 5.25 | -0.21 | 11.22 |  |  |  |
|  |  | 2017t1 | bin644 | bin645 |  |  |  | 7.01 | -0.20 | 13.99 |  |  |  |
|  | *qNL-Chr6-1* | 2019t1 | bin748 | bin749 |  |  |  | 2.64 | 0.20 | 3.71 |  |  |  |
|  | *qNL-Chr10-1* | 2017t1 | bin1100 | bin1101 | 3.24 | 0.09 | 7.10 |  |  |  |  |  |  |
|  | ***qNL-Chr10-2*** | 2017t1 | bin1115 | bin1116 | 4.77 | 0.11 | 10.25 |  |  |  |  |  |  |
|  | *qNL-Chr10-3* | 2017t1 | bin1132 | bin1133 | 3.55 | 0.10 | 8.62 |  |  |  |  |  |  |
|  | *qNL-Chr14-1* | 2017t1 | bin1485 | bin1486 | 2.22 | 0.07 | 4.80 |  |  |  |  |  |  |
|  | *qNL-Chr14-2* | 2017t1 | bin1504 | bin1505 | 3.61 | 0.09 | 7.68 |  |  |  |  |  |  |
|  | *qNL-Chr14-3* | 2017t1 | bin1516 | bin1517 | 2.91 | 0.08 | 6.23 |  |  |  |  |  |  |
|  | *qNL-Chr15-1* | 2017t1 | bin1590 | bin1591 | 2.31 | -0.07 | 4.82 |  |  |  |  |  |  |
|  | *qNL-Chr18-1* | 2019t2 | bin1962 | bin1963 |  |  |  | 2.01 | 0.11 | 4.11 |  |  |  |
|  | *qNL-Chr21-1* | 2017t1 | bin2324 | bin2325 |  |  |  | 3.32 | -0.14 | 6.34 |  |  |  |
|  | *qNL-Chr24-1* | 2019t2 | bin2656 | bin2657 |  |  |  | 2.58 | -0.13 | 5.31 |  |  |  |
|  | *qNL-Chr25-1* | 2017t1 | bin2768 | bin2769 |  |  |  |  |  |  | 11.08 | 2.41 | 1.40 |
|  | *qNL-Chr26-1* | 2017t1 | bin2784 | bin2785 |  |  |  | 2.28 | -0.12 | 4.28 |  |  |  |
| **SH** | *qSH-Chr1-1* | 2019t2 | bin24 | bin25 | 3.50 | 1.05 | 7.42 |  |  |  |  |  |  |
|  | *qSH-Chr1-2* | 2019t2 | bin99 | bin100 |  |  |  |  |  |  | 3.02 | 2.29 | 6.13 |
|  |  | 2019t2 | bin105 | bin106 |  |  |  |  |  |  | 2.38 | 2.06 | 4.86 |
|  | *qSH-Chr4-1* | 2019t1 | bin385 | bin386 | 2.56 | 1.02 | 7.50 |  |  |  |  |  |  |
|  | ***qSH-Chr5-1*** | 2019t1 | bin538 | bin539 | 3.72 | 1.61 | 11.21 |  |  |  |  |  |  |
|  | ***qSH-Chr5-2*** | 2019t1 | bin554 | bin555 | 6.45 | -2.15 | 21.03 |  |  |  |  |  |  |
|  | *qSH-Chr5-3* | 2019t1 | bin640 | bin641 |  |  |  | 4.42 | -1.13 | 8.50 |  |  |  |
|  | ***qSH-Chr6-1*** | 2019t1 | bin750 | bin751 |  |  |  | 6.12 | 1.31 | 11.38 |  |  |  |
|  | *qSH-Chr7-1* | 2019t2 | bin832 | bin833 |  |  |  |  |  |  | 2.69 | 2.32 | 6.58 |
|  | *qSH-Chr11-1* | 2019t2 | bin1229 | bin1230 |  |  |  | 3.31 | -0.66 | 6.35 |  |  |  |
|  | *qSH-Chr11-2* | 2019t2 | bin1243 | bin1244 |  |  |  | 4.36 | -0.76 | 8.25 |  |  |  |
|  | *qSH-Chr11-3* | 2019t2 | bin1267 | bin1268 |  |  |  | 2.85 | -0.63 | 5.76 |  |  |  |
|  | *qSH-Chr12-1* | 2019t2 | bin1278 | bin1279 |  |  |  | 2.09 | -0.52 | 3.83 |  |  |  |
|  | *qSH-Chr12-2* | 2019t1 | bin1351 | bin1351 |  |  |  |  |  |  | 2.56 | -2.81 | 9.12 |
|  | *qSH-Chr14-1* | 2019t1 | bin1522 | bin1523 |  |  |  | 2.08 | -0.75 | 3.65 |  |  |  |
|  | *qSH-Chr16-1* | 2019t1 | bin1737 | bin1738 |  |  |  | 2.17 | -0.75 | 3.61 |  |  |  |
|  | *qSH-Chr18-1* | 2019t2 | bin1918 | bin1919 |  |  |  |  |  |  | 3.72 | 2.48 | 7.58 |
|  | *qSH-Chr19-1* | 2019t2 | bin2054 | bin2055 |  |  |  |  |  |  | 2.20 | -1.92 | 4.57 |
|  |  | 2019t2 | bin2054 | bin2055 | 3.21 | -1.02 | 6.78 |  |  |  |  |  |  |
|  | *qSH-Chr19-2* | 2019t2 | bin2067 | bin2068 |  |  |  |  |  |  | 3.54 | -2.45 | 7.22 |
|  | *qSH-Chr21-1* | 2019t1 | bin2273 | bin2274 |  |  |  |  |  |  | 2.33 | -2.67 | 8.22 |
|  | *qSH-Chr21-2* | 2019t2 | bin2305 | bin2306 |  |  |  | 2.60 | 0.58 | 4.87 |  |  |  |
|  |  | 2019t2 | bin2312 | bin2313 |  |  |  | 3.28 | 0.65 | 6.09 |  |  |  |
|  | *qSH-Chr21-3* | 2019t2 | bin2333 | bin2334 |  |  |  | 2.85 | 0.65 | 6.07 |  |  |  |
|  | *qSH-Chr24-1* | 2019t2 | bin2618 | bin2619 |  |  |  | 2.72 | -0.61 | 5.13 |  |  |  |
|  | *qSH-Chr24-2* | 2019t2 | bin2625 | bin2626 |  |  |  | 3.41 | -0.70 | 6.36 |  |  |  |
|  | *qSH-Chr25-1* | 2019t1 | bin2778 | bin2779 | 2.31 | -1.09 | 8.57 |  |  |  |  |  |  |
| **FW** | *qFW-Chr1-1* | 2018 | bin35 | bin36 |  |  |  |  |  |  | 2.13 | 2.79 | 5.54 |
|  | *qFW-Chr5-1* | 2018 | bin638 | bin639 | 2.78 | -0.01 | 7.83 |  |  |  |  |  |  |
|  | *qFW-Chr23-1* | 2018 | bin2528 | bin2529 |  |  |  |  |  |  | 2.30 | 3.03 | 6.00 |
|  | *qFW-Chr24-1* | 2018 | bin2637 | bin2638 | 1.81 | -0.01 | 3.35 |  |  |  |  |  |  |
| **DW** | *qDW-Chr2-1* | 2018 | bin181 | bin182 | 2.10 | 0.00 | 3.11 |  |  |  |  |  |  |
|  | *qDW-Chr2-2* | 2018 | bin200 | bin201 | 2.01 | 0.00 | 2.99 |  |  |  |  |  |  |
|  | *qDW-Chr3-1* | 2018 | bin319 | bin320 | 2.33 | 0.00 | 3.23 |  |  |  |  |  |  |
|  | *qDW-Chr5-1* | 2018 | bin635 | bin636 | 2.93 | 0.00 | 8.06 |  |  |  |  |  |  |
|  | *qDW-Chr7-1* | 2018 | bin844 | bin845 |  |  |  |  |  |  | 2.35 | -4.47 | 5.97 |
|  |  | 2018 | bin852 | bin853 |  |  |  |  |  |  | 3.02 | -5.17 | 7.60 |
|  | *qDW-Chr7-2* | 2018 | bin874 | bin875 |  |  |  |  |  |  | 2.18 | 4.54 | 5.46 |
|  |  | 2018 | bin882 | bin883 |  |  |  |  |  |  | 2.30 | 4.37 | 5.75 |
|  | *qDW-Chr17-1* | 2018 | bin1891 | bin1892 |  |  |  | 3.01 | 0.00 | 5.58 |  |  |  |
|  | *qDW-Chr21-1* | 2018 | bin2369 | bin2370 |  |  |  | 2.67 | 0.00 | 6.55 |  |  |  |
|  | *qDW-Chr24-1* | 2018 | bin2637 | bin2638 | 3.03 | 0.00 | 5.60 |  |  |  |  |  |  |
|  | *qDW-Chr24-2* | 2018 | bin2667 | bin2668 |  |  |  |  |  |  | 2.73 | -4.35 | 6.85 |
| **GL** | *qGL-Chr1-1* | 2018 | bin1 | bin2 |  |  |  |  |  |  | 2.43 | 3.75 | 6.57 |
|  | *qGL-Chr1-2* | 2018 | bin18 | bin19 |  |  |  |  |  |  | 2.40 | -3.90 | 6.00 |
|  | *qGL-Chr4-1* | 2018 | bin384 | bin385 |  |  |  |  |  |  | 3.75 | 3.65 | 9.59 |
|  | *qGL-Chr4-2* | 2018 | bin465 | bin466 | 3.36 | 0.41 | 8.82 |  |  |  |  |  |  |
|  | *qGL-Chr4-3* | 2018 | bin484 | bin485 |  |  |  | 3.70 | 0.76 | 8.89 |  |  |  |
|  | *qGL-Chr7-1* | 2018 | bin857 | bin858 |  |  |  | 2.45 | 0.60 | 5.76 |  |  |  |
|  | *qGL-Chr17-1* | 2018 | bin1828 | bin1829 |  |  |  | 2.49 | 0.60 | 5.85 |  |  |  |

FER, Field emergence rate; GP, germination potential; GR, germination rate; NL, Number of main stem leaves; SH, seedling height; FW, fresh weight; DW, dry weight; GL, germinal length.

E1, salt stress condition; E2, normal condition; R-value, relative index value dataset. 2017t1, spring of 2017; 2017t2, summer of 2017; 2019t1, spring of 2019; 2019t2, summer of 2019. Figures underlined referred to the common QTLs detected on two datasets on the same year in present study. QTL noted by ‘*’ referred to common QTL detected on two datasets. Bold fonts referred to stable QTL that explained phenotypic variation >10%. Var%, Phenotypic variation explained by a single locus QTL (%). A, Additive effect.
