# Supplementary material for: Identification of candidate genes involved in salt stress response at germination and seedling stages by QTL mapping in upland cotton
Source: G3 (Bethesda). 2022 Apr 26;12(6):jkac099. doi: 10.1093/g3journal/jkac099 (PMC9157077; doi:10.1093/g3journal/jkac099)
Supplement: jkac099_Table_S7 [file jkac099_table_s7.doc]

**Table S7** Pleiotropic loci for salt-tolerant related traits

| **Cluster** | **QTL** | **Year** | **Flanking marker** | | **Under E1** | | | | **Under E2** | | | **R-value** | | |
| --- | --- | --- | --- | --- | --- | --- | --- | --- | --- | --- | --- | --- | --- | --- |
|  |  |  | **L** | **R** | **LOD** | **A** | **Var%** | | **LOD** | **A** | **Var%** | **LOD** | **A** | **Var%** |
| Loci-Chr1-1 | *qGL-Chr1-1* | 2018 | bin1 | bin2 |  |  | |  |  |  |  | 2.43 | 3.75 | 6.57 |
|  | *qGP-Chr1-1* | 2018 | bin4 | bin4 |  |  | |  |  |  |  | 3.46 | 3.40 | 7.98 |
|  |  | 2018 | bin5 | bin6 | 3.23 | 2.97 | 7.68 | |  |  |  |  |  |  |
|  | *qFW-Chr1-1* | 2018 | bin35 | bin36 |  |  |  | |  |  |  | 2.13 | 2.79 | 5.54 |
|  | *qNL-Chr1-1* | 2019t1 | bin37 | bin38 |  |  |  | |  |  |  | 2.04 | 2.55 | 6.44 |
|  | *qGL-Chr1-2* | 2018 | bin18 | bin19 |  |  |  | |  |  |  | 2.40 | -3.90 | 6.00 |
|  | *qSH-Chr1-1* | 2019t2 | bin24 | bin25 | 3.50 | 1.05 | 7.42 | |  |  |  |  |  |  |
| Loci-Chr1-2 | *qFER-Chr1-1* | 2019t2 | bin105 | bin106 | 3.02 | 3.09 | 6.21 | |  |  |  |  |  |  |
|  | *qSH-Chr1-2* | 2019t2 | bin99 | bin100 |  |  |  | |  |  |  | 3.02 | 2.29 | 6.13 |
|  |  | 2019t2 | bin105 | bin106 |  |  |  | |  |  |  | 2.38 | 2.06 | 4.86 |
| Loci-Chr5-1 | ***qSH-Chr5-2*** | 2019t1 | bin554 | bin555 | 6.45 | -2.15 | 21.03 | |  |  |  |  |  |  |
|  | *qFER-Chr5-1* | 2019t2 | bin556 | bin557 |  |  |  | |  |  |  | 3.13 | 11.45 | 6.67 |
|  |  | 2019t2 | bin561 | bin562 |  |  |  | | 2.62 | -2.92 | 4.80 |  |  |  |
| Loci-Chr6-1 | *qNL-Chr6-1* | 2019t1 | bin748 | bin749 |  |  |  | | 2.64 | 0.20 | 3.71 |  |  |  |
|  | ***qSH-Chr6-1*** | 2019t1 | bin750 | bin751 |  |  |  | | 6.12 | 1.31 | 11.38 |  |  |  |
| Loci-Chr7-1 | *qSH-Chr7-1* | 2019t2 | bin832 | bin833 |  |  |  | |  |  |  | 2.69 | 2.32 | 6.58 |
|  | *qDW-Chr7-1* | 2018 | bin844 | bin845 |  |  |  | |  |  |  | 2.35 | -4.47 | 5.97 |
|  |  | 2018 | bin852 | bin853 |  |  |  | |  |  |  | 3.02 | -5.17 | 7.60 |
|  | *qGL-Chr7-1* | 2018 | bin857 | bin858 |  |  |  | | 2.45 | 0.60 | 5.76 |  |  |  |
| Loci-Chr12-1 | *qSH-Chr12-1* | 2019t2 | bin1278 | bin1279 |  |  |  | | 2.09 | -0.52 | 3.83 |  |  |  |
|  | *qFER-Chr12-2* | 2018 | bin1295 | bin1296 |  |  |  | |  |  |  | 2.35 | 4.98 | 4.75 |
| Loci-Chr14-1 | *qNL-Chr14-2* | 2017t1 | bin1504 | bin1505 | 3.61 | 0.09 | 7.68 | |  |  |  |  |  |  |
|  | *qSH-Chr14-1* | 2019t1 | bin1522 | bin1523 |  |  |  | | 2.08 | -0.75 | 3.65 |  |  |  |
|  | *qNL-Chr14-3* | 2017t1 | bin1516 | bin1517 | 2.91 | 0.08 | 6.23 | |  |  |  |  |  |  |
|  | *qFER-Chr14-1* | 2017t1 | bin1523 | bin1524 |  |  |  | | 2.93 | -1.51 | 6.19 |  |  |  |
| Loci-Chr15-1 | *qNL-Chr15-1* | 2017t1 | bin1590 | bin1591 | 2.31 | -0.07 | 4.82 | |  |  |  |  |  |  |
|  | *qFER-Chr15-2* | 2018 | bin1594 | bin1595 | 2.36 | -2.25 | 4.48 | |  |  |  |  |  |  |
| Loci-Chr21-1 | *qSH-Chr21-1* | 2019t1 | bin2273 | bin2274 |  |  |  | |  |  |  | 2.33 | -2.67 | 8.22 |
|  | *qFER-Chr21-1* | 2019t1 | bin2275 | bin2276 |  |  |  | |  |  |  | 1.63 | -6.77 | 5.20 |
| Loci-Chr23-1 | *qFER-Chr23-1* | 2019t2 | bin2524 | bin2525 |  |  |  | |  |  |  | 2.28 | -9.41 | 4.44 |
|  | *qFW-Chr23-1* | 2018 | bin2528 | bin2529 |  |  |  | |  |  |  | 2.30 | 3.03 | 6.00 |
| Loci-Chr24-1 | *qFW-Chr24-1* | 2018 | bin2637 | bin2638 | 1.81 | -0.01 | 3.35 | |  |  |  |  |  |  |
|  | *qDW-Chr24-1* | 2018 | bin2637 | bin2638 | 3.03 | 0.00 | 5.60 | |  |  |  |  |  |  |
| Loci-Chr24-2 | *qFER-Chr24-2* | 2017t2 | bin2644 | bin2645 | 2.41 | 2.38 | 5.00 | |  |  |  |  |  |  |
|  | *qNL-Chr24-1* | 2019t2 | bin2656 | bin2657 |  |  |  | | 2.58 | -0.13 | 5.31 |  |  |  |
| Loci-Chr25-1 | *qFER-Chr25-2* | 2017t1 | bin2753 | bin2754 |  |  |  | | 2.10 | 1.23 | 4.15 |  |  |  |
|  | *qNL-Chr25-1* | 2017t1 | bin2768 | bin2769 |  |  |  | |  |  |  | 11.08 | 2.41 | 1.40 |
|  | *qSH-Chr25-1* | 2019t1 | bin2778 | bin2779 | 2.31 | -1.09 | 8.57 | |  |  |  |  |  |  |
| Loci-Chr26-1 | *qNL-Chr26-1* | 2017t1 | bin2784 | bin2785 |  |  |  | | 2.28 | -0.12 | 4.28 |  |  |  |
|  | *qGR-Chr26-1* | 2018 | bin2790 | bin2791 |  |  |  | |  |  |  | 3.41 | -2.85 | 8.47 |

E1, salt stress condition; E2, normal condition; R-value, relative index value dataset. 2017t1, spring of 2017; 2017t2, summer of 2017; 2019t1, spring of 2019; 2019t2, summer of 2019. Var%, Phenotypic variation explained by a single locus QTL (%). Figures underlined referred to the common QTLs detected on two datasets on the same year in present study. QTL noted by ‘*’ referred to common QTL detected on two datasets. Bold fonts referred to stable QTL that explained phenotypic variation >10%. A, Additive effect.
