# Supplementary material for: Identification of candidate genes involved in salt stress response at germination and seedling stages by QTL mapping in upland cotton
Source: G3 (Bethesda). 2022 Apr 26;12(6):jkac099. doi: 10.1093/g3journal/jkac099 (PMC9157077; doi:10.1093/g3journal/jkac099)
Supplement: jkac099_Table_S10 [file jkac099_table_s10.doc]

**Table S10 Functional description of candidate genes in response to salt stress**

| **Loci** | **Gene ID** | **Gene Name** | **Description** |
| --- | --- | --- | --- |
| Cluster-Chr4-2 | *Gh_A04G1053* | CVA16-2 | V-type proton ATPase 16 kDa proteolipid subunit |
|  | *Gh_A04G1106* | XLG1 | Extra-large guanine nucleotide-binding protein 1 |
|  | *Gh_A04G1046* | Cyp1 | Peptidyl-prolyl cis-trans isomerase 1 |
|  | *Gh_A04G1036* | Utp3 | Something about silencing protein 10 |
| Cluster-Chr5-4 | *Gh_A05G3291* | nagk | N-acetyl-D-glucosamine kinase |
|  | *Gh_A05G3266* | Os03g0733400 | Zinc finger BED domain-containing protein RICESLEEPER 2 |
|  | *Gh_A05G3257* | NA | NA |
|  | *Gh_A05G3246* | NA | Calcium-dependent protein kinase SK5 |
|  | *Gh_A05G3177* | PTB | Polypyrimidine tract-binding protein homolog 1 |
| *qFER-Chr12-3* | *Gh_A12G0415* | NA | Elongation factor 1-gamma |
|  | *Gh_A12G0615* | At2g18630 | UPF0496 protein At2g18630 |
|  | *Gh_A12G0437* | At3g12800 | Peroxisomal 2,4-dienoyl-CoA reductase |
|  | *Gh_A12G0468* | GTE1 | Transcription factor GTE1 |
|  | *Gh_A12G0499* | ntpR | Protein NtpR |
|  | *Gh_A12G0501* | NA | NA |
|  | *Gh_A12G0495* | LBD41 | LOB domain-containing protein 41 |
